# Advancing inorganic electro-optical materials for 5 G communications: from fundamental mechanisms to future perspectives

**DOI:** 10.1038/s41377-025-01851-9

**Published:** 2025-05-12

**Authors:** Hao Wang, Long Chen, Yao Wu, Suwan Li, Guanlong Zhu, Wei Liao, Yi Zou, Tao Chu, Qiuyun Fu, Wen Dong

**Affiliations:** 1 National Demonstrative School of Microelectronics & Wuhan National Laboratory for Optoelectronics & Engineering Research Center for Functional Ceramics of the Ministry of Education, School of Integrated Circuits, Huazhong University of Science and Technology, Wuhan, China; 2 School of Information Science and Technology, Shanghai University of Science and Technology, Shanghai, China; 3 College of Information Science and Electronic Engineering, Zhejiang University, Hangzhou, China

**Keywords:** Optical materials and structures, Electronics, photonics and device physics

## Abstract

In the 5 G era, the demand for high-capacity and fast fiber-optic communication underscores the importance of inorganic optical materials with high electro-optical (EO) coefficients, rapid responses, and stability for efficient electro-optical modulators. The exploration of novel EO materials and their applications remains in the early stages. At present, research mainly focuses on the performance of EO materials and devices. However, the EO coefficients of different preparation methods for the same material and different materials vary significantly. Currently, a crucial gap lies in understanding the link between the EO effect and ferroelectric polarization, hindering advancements in ferroelectric material optimization. This article offers a comprehensive insight into the EO effect, initially discussing ferroelectric polarization and its relationship to the phenomenon. It then reviews standard inorganic ABO_3_ metal oxide ferroelectric ceramics and thin films, followed by an examination of emerging ferroelectrics such as HfO_2_-based polymorph ferroelectrics and ZnO/AlN-based materials. The article concludes by addressing the challenges in investigating ferroelectric EO mechanisms and provides an outlook on the future of EO material research, including a review of the latest developments in EO effect mechanisms and their optimization for light modulation, as well as an exploration of potential areas for high-performance EO materials research.

## Abstract (web summary)

This review highlights the advancement of inorganic electro-optical materials. It underscores strategies for optimizing performance through multiscale analysis and design, offering guidance for developing high-performance electro-optic devices.

## Graphical abstract

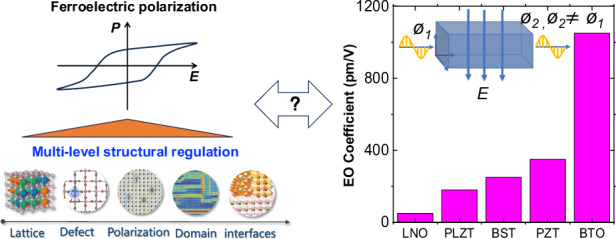

## Introduction

Optical communication excels with its extensive transmission range, massive data-carrying capacity, and unparalleled speed, making it an indispensable pillar for meeting the needs of artificial intelligence and big data processing as well as constructing the backbone network, bearer networks, and inter-base station connections in 5 G infrastructure^1,2^. By harnessing light as its carrier wave, optical communication modulates user information onto light waves, seamlessly transmitting them through media like the atmosphere or optical fibers, ultimately to be decoded and reconstructed by optical receivers^3,4^. At the core of this process, electro-optical (EO) modulators play a vital role, necessitating meticulous attention to their manufacturing procedures and material selection. Over time, EO modulators have evolved into distinct categories based on the electrode materials used in their substrates, including silicon-based photoelectronic modulators, lithium niobite-based EO modulators, and innovative ferroelectric EO modulators.

**Fig. 1:**
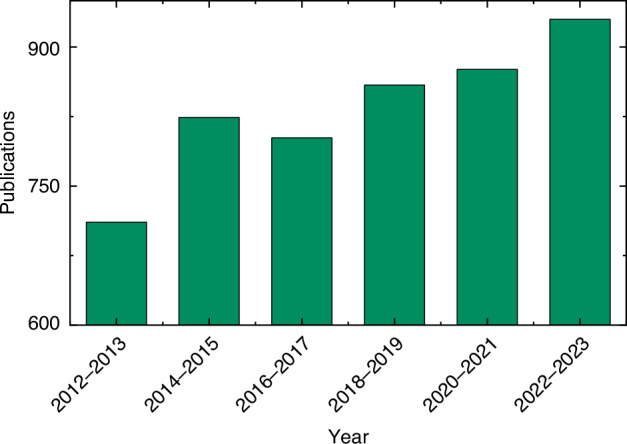
The publications in recent years. (Collected from Web of Science, search “ferroelectric/electro-optic effect”)

Currently, the application domains of these materials demand stringent criteria, including high bandwidth, minimal optical loss, low electrical power consumption, and a high signal-to-noise ratio. Unfortunately, traditional silicon-based, III-V, and compound-based modulators fall short of simultaneously fulfilling these requirements. Despite lithium niobate’s esteemed position in the optoelectronics industry for its exceptional crystal properties, its limitations in processing complexity, doped waveguide structure, bulkiness, high cost, constrained bandwidth, and elevated driving voltage hinder its suitability^5–7^. On the other hand, thin-film lithium niobate (LiNbO_3_, LNO) shows promising prospects despite its relatively recent development. Its technology and performance still have ample room for growth, necessitating further research and iterative development. Emerging ferroelectric EO materials, such as BaTiO_3_ (BTO), PbZr_1-*x*_Ti_*x*_O_3_ (PZT), and La-doped PZT (PLZT), also show significant potential for further development. However, structurally, thin-film materials outperform crystalline and transparent ceramics. This is attributed to the latter’s intricate processing challenges and cost-effectiveness, implying that thin-film materials are poised for broader development prospects^8,9^. Furthermore, as evidenced by Fig. 1, the realm of ferroelectricity has emerged as a prominent area of research in recent times, with an increasing number of studies dedicated to exploring the effects and materials associated with this phenomenon.

**Fig. 2:**
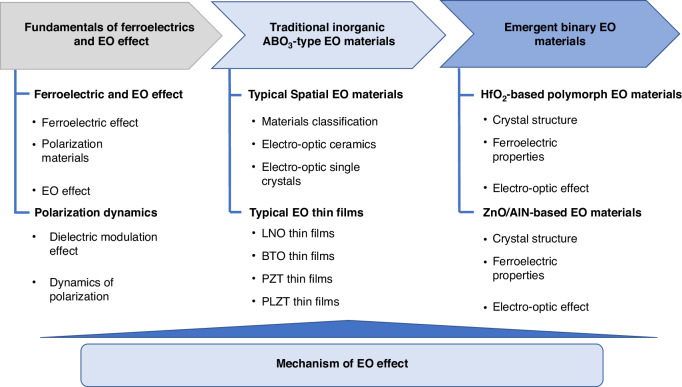
Outline of the review

As future science and technology advance at a breakneck pace, there is a mounting demand for optical communication systems to deliver data at faster speeds and greater capacities, catering to the diverse requirements of various sectors anticipating swifter and more substantial data transmissions. EO modulators play a pivotal role in fiber-optic communication, and the evolution of this technology necessitates modulators that offer quicker modulation rates, reduced losses, and a more compact form factor. Presently, despite the abundance of inorganic ferroelectric material types and research endeavors, there remains a gap between the production and widespread deployment of practical devices. Each ferroelectric EO modulator possesses unique strengths and limitations. As shown in Fig. 2, this review provides an in-depth exploration of the ferroelectric and EO properties of several exemplary inorganic ferroelectric EO materials. It discusses the multiscale polarization structures within ferroelectrics and their fundamental dynamic behaviors, as well as the interplay between domain structure, dielectric tunability, refractive index, and the EO effect. The article also addresses the current challenges, particularly the elusive link between domain structure and refractive index, within the realm of potential next-generation inorganic EO materials. The primary obstacle we discovered stems from the intricate and multifaceted polarization structures in ferroelectrics, which complicate the establishment of a precise quantitative relationship between ferroelectric polarization and the EO effect. Ultimately, the article outlines future development trajectories and identifies promising avenues for the advancement of these materials.

## Fundamentals of ferroelectric and EO effect

### Ferroelectric materials and polarization

**Fig. 3:**
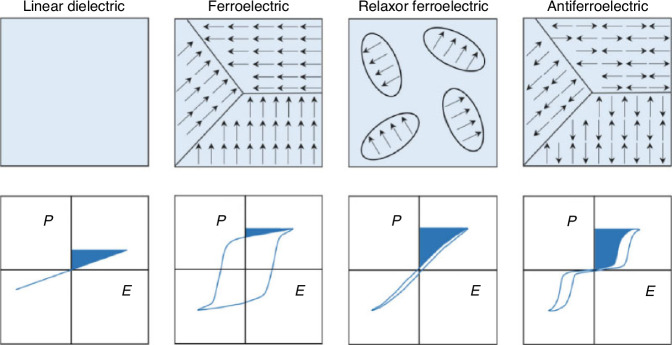
Schematic illustration of dipoles, domain structures, and polarization hysteresis loops for linear dielectrics, ferroelectrics, relaxor ferroelectric, and antiferroelectric materials. The shaded area represents the recoverable energy density of dielectric and ferroelectric capacitors throughout positive charge-discharge cycles. Reproduced with permission from ref. ^10^

In general, as shown in Fig. 3, based on the varying responses of polarization to an applied electric field, dielectric materials can be classified into linear dielectrics, classical ferroelectrics, relaxor ferroelectrics, and antiferroelectrics (AFEs)^10^. Ferroelectricity, characterized by reversible electric polarization due to ionic displacement, was experimentally discovered a century ago, sparking research into its fundamental properties and potential applications^11–14^. Here, ferroelectrics, such as BTO, are generally referred to as classical ferroelectrics with microscale domain structures. Meanwhile, relaxor ferroelectrics, such as PZT, show nanosized polar domains and generally higher piezoelectricity with easier polarization switching compared with classical ones due to their generally lower switching barriers between the two stable polarization states. In relaxor ferroelectrics, nanoscale domains or polar nanoregions (PNRs) modulate the domain wall configuration, resulting in a macroscopic reduction of hysteresis, evident as a thin hysteresis loop in Fig. 3. In contrast to the classical hysteresis loop depicted in Fig. 3, which exhibits negligible hysteresis, The observed nonlinearity is clearly related to the presence and movement of polarization within the nanoscale domains^15^.

**Fig. 4:**
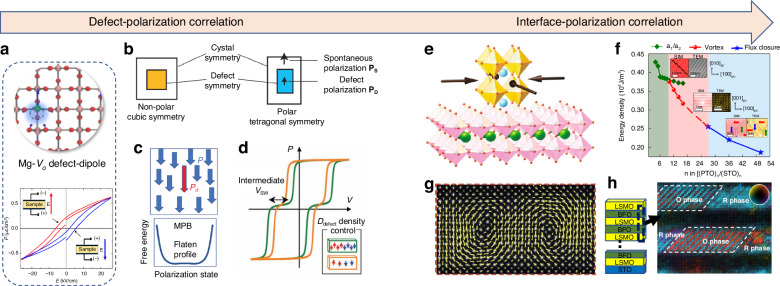
Multiscale structures in ferroelectrics in relation to defects and interface. **a** The local structure around the Mg_Ti_ -*V*_*o*_ complex in 0.25 at% Mg-doped rutile TiO_2_ modeled with density functional theory_._ Reproduced from ref. ^18^ with permission from Royal Chemistry Society: Materials Horizons. **b** Symmetry-conforming property of point defects of the polar tetragonal symmetry compared with the non-polar cubic symmetry. Reproduced from ref. ^23^ with permission from Springer Nature: Nature Materials. **c** A schematic of the heterogeneous polar states of a ferroelectric, where arrows represent the host polarization *P* and defect-induced polarization *P*_*d*._ Reproduced from ref. ^19^ with permission from John Wiley and Sons: Advanced Materials. **d** Vary of the intermediate switching voltage according to the density of the aligned defect. Reproduced from ref. ^21^ with permission from John Wiley and Sons: Advanced Materials. **e** A schematic of an oxide superlattice epitaxially grown on an underlying substrate. Reproduced from ref. ^24^ with Springer Nature: Nature Review Materials. **f** Phase diagram and total energy density for the (PTO)n/(STO)_*n*_ superlattice grown on the DyScO_3_ substrate calculated by the phase-field simulations and experiments. Reproduced from ref. ^28^ with permission from the American Chemical Society. **g** TEM image of the polar vortices. Reproduced from refs. ^30^ with permission from Springer Nature: Nature. **h** TEM image of the BFO/LSMO superlattice. Reproduced from ref. ^26^ with permission from the American Chemical Society

It is well known that ferroelectric polarization is mainly decided by the phase structure and domain structure intrinsically, however, external structural factors, such as point defects, interface, have been widely used to regulate and optimize the macroscopic polarization^16,17^. As shown in Fig. 4a–d, point defect with local defect-dipolar effect prototypically exampled in 0.25 at% Mg-doped TiO_2_ where significant displacement of Ti and O (blue arrow) can be induced due to local symmetry breaking^18^. *P–E* loops of Mg-doped rutile TiO_2_ collected after poling with a negative electric field E (red) and a relatively positive electric field (blue). A poling electric field of 2.5 kV/cm was applied for a certain time on the sample at room temperature. Therefore, the point defect-dipolar effect is assumed to break the long-range order and couple with the intrinsic polarization for enhanced polarization switching, ferroelectricity, and piezoelectricity, due to a flattened free energy profile^19–23^. Besides point defects, the interface can manipulate the thermal dynamic variables to unleash the properties of the hidden phase by altering the delicate balance between competing spin, charge, orbital, and lattice degrees of freedom^24–27^. For example, the superlattice interface (Fig. 4e) engineered stability of polar vortex polarization structure (Fig. 4f)^28,29^ where the top left shows the simulation and planar TEM result of in-plane view of *a*_*1*_*/a*_*2*_ twin-domain structure for *n* = 6. The middle left and right are the vortex structures for *n* = 10 from simulation and experimental TEM mapping, respectively. The bottom left (Fig. 4g) and right insets are the cross sections of flux-closure structure for *n* = 50 from phase-field simulation and experimental TEM vector mapping, respectively^30^. “SIM” and “TEM” stand for simulation and transmission electron microscopy data, respectively. Similarly, emergent strain-induced antipolar phase BiFeO_3_ − La_0.7_Sr_0.3_MnO_3_ (BFO/LSMO) superlattice can be observed (Fig. 4h)^26^. Therefore, defects and interface, together with the lattice, domain structure, established the main multiscale structures within the ferroelectrics. The emergent polarization state and domain structures further enrich the properties of ferroelectric polarization.

### EO effect

**Fig. 5:**
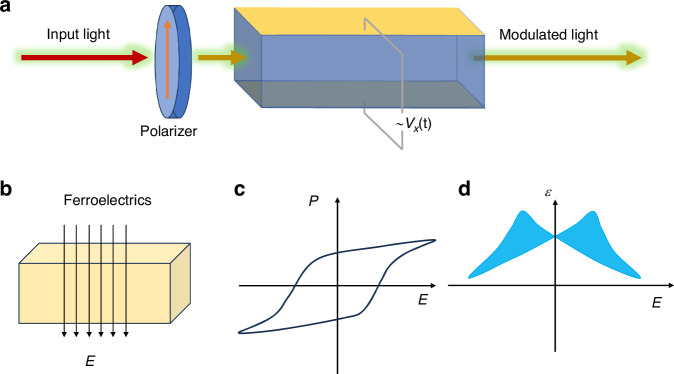
EO effect and ferroelectric polarization. **a** Schematic illustration of the EO modulator where the phase of the output light is modulated by the ferroelectrics with externally applied electrodes, **b** Illustration of the polarization response to the external electric field. Closed-loop polarization *P*, **c** and dielectric constant *ε*, **d** hysteresis under the external electric field *E* in ferroelectrics

Take the space EO modulators as an example (Fig. 5a). This voltage change induces a change in the refractive index of the crystal, thereby varying the phase of a linearly polarized laser beam traversing it. The EO effect is a phenomenon wherein certain isotropic, transparent materials exhibit optical anisotropy upon the application of an electric field, resulting in a modulation of their refractive index. These crystals, known as EO crystals, display a linear relationship between the change in refractive index and the intensity of the applied electric field, a phenomenon termed the linear EO effect or Pockels effect. Alternatively, the second EO effect, also known as the Kerr EO effect, exhibits a proportionality to the square of the applied electric field intensity. Mathematically, the variation in a crystal’s refractive index as a function of the applied field strength can be elegantly expressed as^31^:

1 $$\,n={n}_{0}+a{E}_{0}+b{E}_{0}^{2}+\cdots$$

where the constants *a* and *b* represent the linear EO coefficient and the secondary EO coefficient of the crystal, respectively, while ${n}_{0}$ denotes the refractive index of the crystal in the absence of an electric field. The refractive index of the crystal can be conceptually linked to the luminous body, and the alterations in the refractive index induced by the electric field can be interpreted as modifications in the shape, size, and orientation of this luminous body:

2 $$\Delta {\beta }_{{ij}}={\beta }_{{ij}}-{\beta }_{{\rm{i}}j}^{0}={r}_{{ijk}}{E}_{k}\,+\,{h}_{{ijpq}}{E}_{p}{E}_{q}+\cdots$$

where the ${\beta }_{{ij}}$ represents the dielectric impermeability tensor, essentially the inverse of the dielectric constant at optical frequencies. Meanwhile, the ${r}_{{ijk}}$ and ${h}_{{ijpq}}$ stand as the primary and secondary EO coefficients, respectively. *i*, *j*, and *k* are used here to represent the components of tensors, which are indices in multidimensional arrays used to describe physical quantities in different directions. Current research endeavors predominantly concentrate on the linear EO effects, as opposed to the secondary ones. The linear EO behavior of crystals can be comprehensively characterized and quantitatively assessed through the utilization of the following matrices:

3 $$\,\left[\begin{array}{c}\Delta {\beta }_{1}\\ \Delta {\beta }_{2}\\ \Delta {\beta }_{3}\\ \Delta {\beta }_{4}\\ \Delta {\beta }_{5}\\ \Delta {\beta }_{6}\end{array}\right]=\left[\begin{array}{c}{\beta }_{1}-{\beta }_{1}^{0}\\ {\beta }_{2}-{\beta }_{2}^{0}\\ {\beta }_{3}-{\beta }_{3}^{0}\\ {\beta }_{4}\\ {\beta }_{5}\\ {\beta }_{6}\end{array}\right]=\left[\begin{array}{ccc}{r}_{11} & {r}_{12} & {r}_{13}\\ {r}_{21} & {r}_{22} & {r}_{23}\\ {r}_{31} & {r}_{32} & {r}_{33}\\ {r}_{41} & {r}_{42} & {r}_{43}\\ {r}_{51} & {r}_{52} & {r}_{53}\\ {r}_{61} & {r}_{62} & {r}_{63}\end{array}\right]\left[\begin{array}{c}{E}_{1}\\ {E}_{2}\\ {E}_{3}\end{array}\right]$$

where the subscript of $\Delta {\beta }_{{ij}}$ and ${\beta }_{{ij}}$ are abbreviated as $\Delta {\beta }_{i}$ and ${\beta }_{i}$ for convenience after matrix multiplication operation.

In non-central materials, both the linear and secondary EO effects coexist. Researchers in the field of programmable and reconfigurable photonic circuits find these effects particularly intriguing due to their distinct EO responses, enabling the conception of varied device architectures.

### Dielectric modulation effect

Ferroelectrics constitute a unique class of materials characterized by spontaneous polarization, where the orientation of their polarization vector can be reoriented by an external electric field. These materials not only exhibit hysteresis loops but also prominent EO effects when an electric field is applied. The spontaneous polarization energy inherent in ferroelectrics imparts macroscopic properties akin to those of an electric dipole to the crystal^32^. Notably, this polarization can be redirected in response to an external electric field. The correlation between ferroelectric polarization dynamics and the EO effect is a complex relationship underlying the EO mechanism. It involves how changes in the polarization of a ferroelectric material can influence the refractive index of an optical medium, leading to variations in the phase, intensity, or polarization state of light passing through it. Ferroelectric materials are characterized by their ability to exhibit a permanent electric polarization that can be reversed by applying an external electric field. This polarization reversal often accompanies domain switching, which is the reorientation of microscopic regions (domains) within the material to align with the applied field. The dynamics of this process—how quickly and through what mechanisms the domains switch—can have a direct impact on the EO properties of the material.

This long-range ordered ionic displacement forms polar regions with polarization vectors in space, creating ferroelectric domains at the microscopic level. Within the same domain, the alignment direction of all-electric dipole moments is consistent, whereas the direction of polarization vectors may vary in different areas. The mutual coupling between domains forms relatively stable domain configurations, including traditional ferroelectric domains such as single domains, 180° domains, and 90° domains (e.g., a/c domains)^33^. This configuration of ferroelectric domains and the corresponding ferroelectric polarization changes with variations in external electric field, temperature, and stress.

**Fig. 6:**
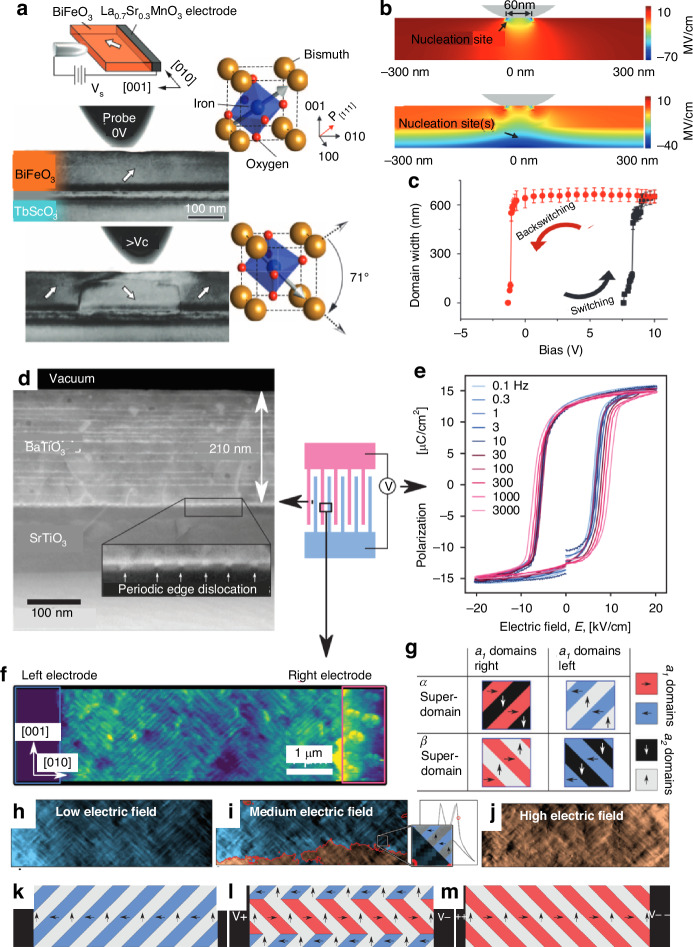
Domain tuning behaviors in out-of-plane and in-plane electric field. **a** Cross-sectional TEM image of the BiFeO_3_ film before and after switching by a 4 V bias. Switching occurs by 71° rotation of the polarization beneath the tungsten tip. **b** Normal component of the electric field from the surface probe to the thin film from top and bottom Schottky junctions. **c** Hysteresis loop of the domain width as a function of bias. Reproduced from ref. ^34^ with permission from American Association for the Advancement of Science. **d** Annular dark field (ADF) STEM image of the 210 nm BaTiO_3_ film; Periodic edge dislocations at the interface are highlighted in the inset. **e** Polarization-electric field hysteresis loops from 0.1 Hz to 3 kHz. **f** Conventional PFM image (driving voltage applied to the tip). *a*_2_ domains appear as diagonal bright lines separated by dark *a*_1_ domains. **g** Types of superdomains in an *a*_1_/*a*_2_ domain structure. **h**–**j** Evolution of the domain structures as a function as the electric field increases to the high electric field. **k**–**m** Schematic illustration of the domain evolution corresponding to (**h**–**j**). Reproduced with permission from ref. ^39^

As shown in Fig. 5b–d, the ferroelectric polarization macroscopically forms both polarization and dielectric hysteresis loops under the modulation of an external electric field. This includes closed-loop polarization hysteresis loops and “butterfly-shaped” strain/dielectric constant hysteresis loops. A hysteresis loop relationship exists between the polarization intensity of ferroelectrics and the applied electric field, serving as a crucial indicator of ferroelectricity. It is this similarity in hysteresis loops, reminiscent of those found in ferromagnetic materials, that lends the name “ferroelectric” to this class of materials. From a structural point of view, the domain switching behavior behind the hysteresis loop under out-of-plane and in-plane electric fields, respectively, are different. For example, Fig. 6 shows the difference between the domain switching mechanisms between out-of-plane electric field tuning and in-plane electric field tuning.

For case with out-of-plane electric field, Fig. 6a shows the schematic and corresponding cross-sectional TEM image of the BiFeO_3_ thin films with bottom electrode while the PFM tip serves as the top electrode^34^. The TEM image shows that a domain nucleation behavior dominates the domain switching process. Figure 6b shows the normal component of the electric field from the surface probe (up) and. including top and bottom Schottky junctions (down). The out-of-plane electric field, *E*_*Z*_, for this tip geometry at a −2V bias, as calculated by finite element analysis, is predominantly concentrated at the free surface. Upon the Schottky barriers of 0.85 V and 0.77 V, originating from the junctions of n-type BiFeO_3_ with La_0.7_Sr_0.3_MnO_3_ and tungsten respectively^35^, the total build-in electric field *E*_*Z*_ field distributions can be calculated (Fig. 6c). A pronounced band along the La_0.7_Sr_0.3_MnO_3_ interface emerges, where polarization nucleation is likely to occur due to a strong negative field directed towards the substrate. This observation aligns with TEM findings. Despite the inability to probe the electrical properties of the Schottky junctions since conduction was bulk-limited, they remain a prevalent feature of planar ferroelectric oxides^36,37^. These junctions are recognized for determining nucleation sites between symmetric planar electrodes in BiFeO_3_ films^38^. Variations in the relative strengths and distributions of these built-in fields can arise based on assumptions about Schottky barrier heights, depletion width, and electrode geometry. Nevertheless, the built-in fields presented in Fig. 6c are of comparable magnitude to the tip field and should not be overlooked.

For the case with an in-plane electric field, Agar et al. reported a high velocity, low-voltage collective in-plane switching in (100) BaTiO_3_ thin films^39^. Scanning transmission electron microscopy (STEM) images reveal individual layers at the interface (Fig. 6d), where epitaxial strain is relaxed by edge dislocations. Piezoforce microscopy (PFM) studies show a_1_/a_2_ superdomains with alternating polarization domains along the (010) and (001) in-plane axes, and 90° domain walls along the (011) directions (Fig. 6e, f). Interdigitated electrode measurements of in-plane ferroelectric hysteresis loops show sharp, square loops with switching frequencies from 0.1 Hz to 3 kHz (Fig. 6g), corresponding to a domain wall velocity of ≈500 cm s⁻¹. Surprisingly, this exceeds velocities observed in BaTiO₃ single crystals at the coercive field^40^, despite strong clamping effects that typically suppress irreversible domain wall motion in epitaxial thin films^23,41,42^. This behavior is attributed to the strongly correlated domain structure. Both theory and experiment indicate that the in-plane electric field concentrates at the electrode edges, facilitating nucleation before reaching the coercive field^43^. Therefore, the strong collective switching behavior with in-plane electric field differs from that of the nucleation behavior with out-of-plane electric field (Fig. 6h–j).

From a device standpoint, in-plane polarization is advantageous because it maintains the polarization within the plane where the substrate enforces elastic boundary conditions, resulting in stronger collective behavior. Tensile strain can generate various dense in-plane domain configurations^44,45^, whereas compressive strain leads to monodomain states or domain patterns that combine both in-plane and out-of-plane elements. Therefore, domain polarization reversal is not only related to the direction of the modulation electric field but also to the direction of stress received during film growth.

In recent years, advancements in material property modification and deeper investigations into ferroelectric polarization microstructures have led to the discovery of smaller-scale ferroelectric domains, such as PNR in relaxor ferroelectrics^46,47^. The new localized nanodomain structures are becoming smaller in terms of morphology and scale, which presents both opportunities and challenges. The miniaturization of localized nanodomains means that the polarization-switching dynamics of domain structures are expected to be maintained at higher frequencies.

### High-frequency dynamics of polarization domain

**Fig. 7:**
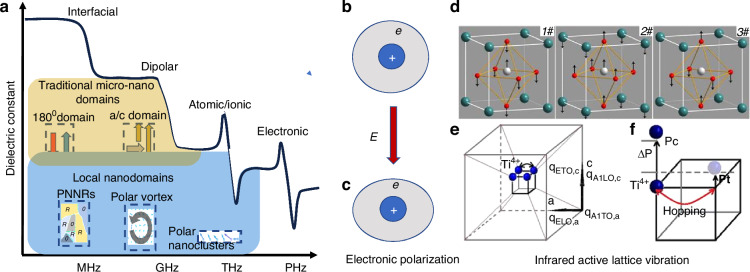
Polarization mechanism and dynamics. **a** Frequency characteristics of dielectric constant in relation to the domain structures at different scales. The upper part of the figure provides a schematic diagram of the dielectric polarization response spectrum of traditional ferroelectric domains below GHz (indicated by gray dashed lines and gray text). The lower part of the figure presents a schematic diagram of the dielectric response spectrum of localized nanodomains in the THz frequency range (indicated by red dashed lines and red text). **b**, **c** Schematic illustration of the electric field-induced variation of electronic polarization arising from the electron cloud around the ion nucleus. **d** Eigen-lattice vibrations of BTO: (1#) Slater, (2#) Last, and (3#) Axe modes. **e** Schematic diagram of order-disorder feature, i.e., Ti cation hopping at four symmetry equivalent positions (along (111) direction) in the displacive plane, the propagating direction of *A*_1_(TO, LO) and *E*(TO, LO) modes, the ferroelectric axis is along *c* direction. **f** The schematic diagram for evaluating the magnitude of the relaxation frequency. Reproduced from ref. ^49^ with permission from the American Institute of Physics Publishing

Figure 7a Frequency characteristics of dielectric constant in relation to the domain structures at different scales. The upper part of the figure provides a schematic diagram of the dielectric polarization response spectrum of traditional ferroelectric domains below GHz (indicated by gray dashed lines and gray text). The lower part of the figure presents a schematic diagram of the dielectric response spectrum of localized nanodomains in the THz frequency range (indicated by red dashed lines and red text).

The dielectric response is intrinsically linked to the polarizable structure or element within the crystals. As shown in Fig. 7a, traditional ferroelectric domain structures, such as a/c domains, 180° domains, etc., mostly range from several tens of nm to µm in size, with most polarization response frequencies below GHz, making it difficult to exhibit significant dielectric polarization responses at THz or higher frequency range, let alone a real 1500 nm optical wavelength electric field^13^. Typically localized nanodomains, such as PNRs, polar vortices, and polar nanoclusters, have relative scales reaching sub-10 nanometers or even a few lattice constants, allowing for response frequencies that are higher than those of traditional ferroelectric domains. For example, polar vortices are proved to show collective THz vibration dynamic behavior, but it is still difficult to be active at optical frequency^48^.

Therefore, the dielectric response at optical frequency intrinsically comes from the electronic polarization (Fig. 7b, c) and infrared lattice vibration mode. Take the BTO as an example, for the tetragonal phase, a structural model can be proposed as shown in Fig. 7d, where Ti cations displace along the 〈111〉 directions and hop between four equivalent positions along the 〈100〉 or 〈110〉 directions (Fig. 7e, f)^49^. This phenomenon is crucial for understanding the dielectric behavior of BTO, which exhibits both displacive and order-disorder features. The propagation direction of the A1-E pair modes, specifically the A1(TO, a) mode, is along the a-axis and couples with the hopping of Ti cations because both vibrate in the same plane with correlated Ti cations. Consequently, the A1 soft mode interacts with the order-disorder mode of Ti cations. This coupling between the A1 soft mode and the order-disorder feature leads to discrepancies in the c-axis permittivity observed experimentally compared to that predicted by soft mode theory. Regarding E(TO) modes along the c-axis, they do not couple with Ti cation hopping.

**Fig. 8:**
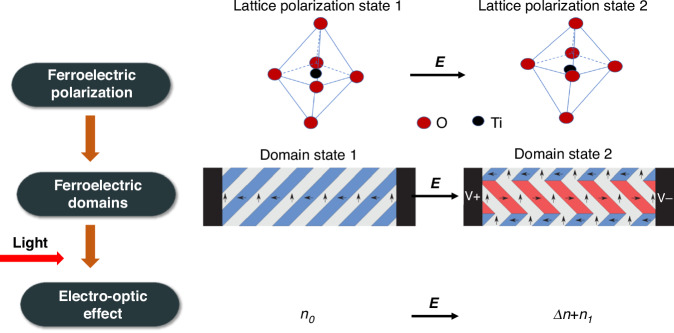
The EO effect in relation to the polarization tuning. Illustration of the change of the domain state1 to domain state 2 in BTO thin films under electric field *E*. Reproduced with permission from ref. ^39^. The electric field change *ΔE* leads to the change of the domain structure. The polarization state changes from state 1 to state 2, which changes the lattice polarization state and refractive index

It is obvious that the domain polarization is not directly involved in the dielectric response at optical frequency. However, why the modulation of the domain polarization still be an important part of the EO effect in ferroelectrics? The EO effect refers to the change in refractive index when an electric field is applied. This effect is directly seen from the variation of refractive index Δ*n* under the tuning electric field Δ*E*. As shown in Fig. 8, here’s how the correlation works:

#### Polarization dynamics

When an external electric field is applied to a ferroelectric material, the polarization and lattice begin to change as domains reorient. The speed and mechanism of this reorientation (e.g., domain wall motion or rotation of polarization vectors) determine the temporal response of the polarization. Under an external electric field, the domain structure changes, leading to two polarization states. Experimental and theoretical investigations have conclusively demonstrated that the dynamics of the ferroelectric domain wall significantly impact hysteresis characteristics^32,50^. The polarization states change the polarizable units within the crystal lattice, leading to the change in polarization and refractive index.

#### Refractive index change

The refractive index is structurally linked to the domain structure and dynamically linked to the polarization state of the lattice. The changing polarization alters the electric field distribution inside the material, which in turn affects the refractive index via the EO effect. The magnitude and rate of this refractive index change depend on the polarization dynamics. Theoretically, the ferroelectric polarization affects the refractive index in the form of strain or inverse piezoelectric effect^51,52^.

#### Optical response

Light passing through the material experiences changes in its phase, intensity, or polarization state due to the varying refractive index. For example, if the refractive index changes uniformly across the material, this might result in a phase shift of the transmitted light, which is the basis for many EO modulators.

The essential functional components of ferroelectric materials encompass crystal lattices, defects, electrical domains, ferroelectric polarization, heterogeneous interfaces, among others, exhibiting polarizations across various hierarchical structures. At a wavelength of 1550 nm, the Pockels tensor is primarily governed by the displacement polarization of lattice ions and the polarization of electron clouds within these intricate multi-level architectures. Furthermore, the orientation of the ferroelectric polarization domain modulates the polarization state of light, while the reversal of ferroelectric polarization directly influences the displacement polarization of lattice ions.

However, since the domain structure and its corresponding ferroelectric polarization change under an external field, the optical frequency polarization response will change due to the polarization-induced lattice strain. Therefore, the interplay between ferroelectric polarization dynamics and the EO effect is a critical factor in understanding and designing devices that use these materials for optical modulation and other EO applications. The efficiency and speed of these devices are directly influenced by the underlying ferroelectric properties and how they evolve under applied electric fields. The ferroelectric properties are intrinsically decided by the composition and phase of the ferroelectric materials types. In the following, we will introduce typical inorganic EO materials.

## Overview of inorganic ABO_3_-type optical materials

The development of EO materials has seen key milestones over the past century. Ferroelectricity was first experimentally discovered a century ago, leading to Valasek’s 1920 study on Rochelle salts showing their ferroelectric domains under polarized light. This led to the 1935 discovery of KDP by Scherrer and Busch^53^, but the application is limited due to its fragility and water solubility^54^. The 1950s brought the discovery of BTO by the Titanium Alloy Manufacturing Company, expanding the use of ferroelectric materials^11,55^.

The confirmation of ferroelectricity in inorganic materials sparked a surge of interest in perovskite oxides, ultimately unraveling novel materials like the PZT composition, now pivotal in piezoelectric applications, and BTO derivatives utilized in capacitors. In 1970, Haertling G.H. revolutionized the field by crafting transparent EO ceramic PLZT through ball milling and hot-press sintering, positioning PLZT as a focal point in EO material research^56^. Rolf Landauer’s 1976 insight into the existence of a free energy barrier in ferroelectric switches, implying a region of negative local curvature and theoretically negative capacitance segments in the free energy landscape^57^, further propelled advancements. Most notably, in 2014, negative capacitance was reported to amplify voltage in ferroelectric thin films, offering a promising avenue for ferroelectric EO material research^58^.

Throughout the evolution of EO materials, the journey from the seminal discovery of ferroelectricity in 1920 to the groundbreaking development of transparent ceramics in 1970 underscores the remarkable advancements achieved in the 20th century within the realm of ferroelectric EO materials. Echoing this progress, the editors of Nature Materials highlighted in 2020 the relentless pace of research and application of these systems, fueled by the initial analogy and Valasek’s pivotal observation of hysteresis. In the second century, ferroelectricity is anticipated to be equally prolific and groundbreaking as its predecessor^11^.

The fundamental principle behind silicon-based photoelectron modulation lies in the free carrier plasma dispersion effect, whereby precise control over the concentration of free carriers dictates changes in refractive index. In a recent 2023 study, Yu et al. comprehensively reviewed the performance of various silicon-based photonic modulators, showcasing maximum data rates spanning from 40 to 240 Gbps, albeit with losses typically exceeding 3 dB^59,60^. In stark contrast, state-of-the-art lithium niobite thin-film EO modulators have achieved remarkable modulation rates of 320 Gbps while maintaining losses below 1 dB^61^. However, silicon-based optoelectronic modulators face limitations, including relatively bulky structures, high modulation voltages, and inferior integration capabilities compared to advanced EO modulators. Furthermore, their modulation linearity suffers due to inherent material and structural constraints, emphasizing the pressing need for improvements in this area to enhance silicon-based EO performance^62^.

**Fig. 9:**
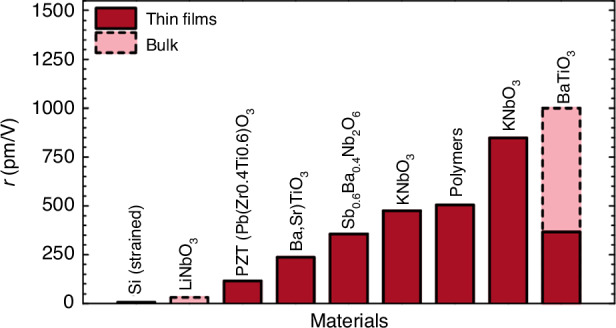
Comparison of the effective Pockels coefficients *r* of common bulk and thin-film nonlinear materials. Reproduced from ref. ^64^ with permission from IEEE: Journal of Lightwave Technology

LNO and other ferroelectric-based EO modulators harness the Pockels EO effect, enabling the refractive index of EO materials to be modulated by an applied electric field. In comparison to silicon-based modulators, these EO material-based modulators boast lower loss, higher modulation rates, superior linearity, and compact sizes, thereby becoming the preferred choice for optical wave modulation in fiber-optic communication systems. Common EO materials encompass III-V compound materials, silica-based materials, polymers, and inorganic ferroelectric materials, each with its unique merits and drawbacks. III-V compounds, for instance, suffer from high optical transmission losses and significant costs, while polymers are cost-effective but lack stability and mechanical strength. Silicon-based silica poses stringent processing requirements^63^. Inorganic ferroelectric materials, on the other hand, exhibit fewer defects, large EO coefficients, swift response times, and robust stability, making them ideal candidates for EO modulator waveguide materials. Presently, LNO, BTO, and PZT are the primary inorganic optical materials in use. Notably, as shown in Fig. 9, the new ferroelectric EO materials surpass lithium niobite in aspects such as size and power consumption^64^, with EO coefficients reaching as high as 1000 pm/V^65,66^, compared to lithium niobite’s ~1 pm/V^67^. From the results, it is obvious that different ferroelectric materials can show significant magnitude level differences in their EO coefficients. In the following, we will describe the ferroelectric properties and EO effect for some typical ferroelectric materials.

### Typical space EO materials

#### EO ceramics

EO ceramics constitute a diverse array of ceramic materials renowned for their EO effect. Their unparalleled physical attributes, including remarkable hardness, outstanding resistance to chemical attack, and superior heat tolerance, have facilitated their extensive application across various industries^68^. Notably, most EO ceramics possess light transmittance unmatched by conventional ceramics, and their refractive index undergoes electric field-induced changes during phase transitions, yielding the electronically controlled birefringence effect. This unique property finds application in optical modulators, memory devices, photoelectric sensors, spectral filters, optical switches, electrically activated multi-color displays, optical valves, and memory elements, among others.

**Fig. 10:**
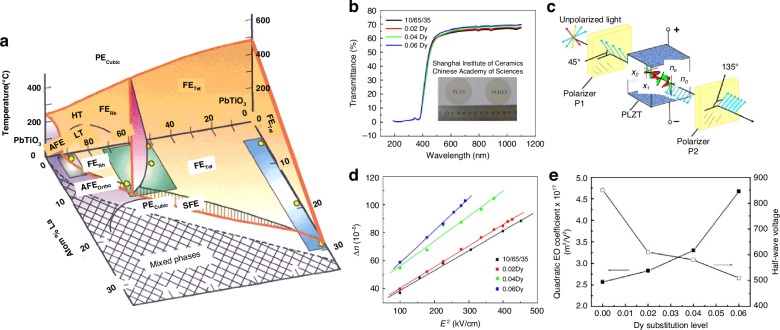
Ferroelectric and EO properties of PLZT material. **a** Room temperature phase diagram of PLZT system. Reproduced from ref. ^74^ with permission from John Wiley and Sons: Journal of American Ceramic Society. **b** The transmittance spectra of PLDZT ceramics are presented as a function of Dy substitution, with an inset featuring a photograph of both PLZT and PLDZT transparent ceramics. **c** Basic setup for evaluating electro-optic modulator characteristics. **d** The relationship between birefringence and the quadratic electric field in PLDZT ceramics is explored, with a focus on its dependence on Dy substitution. **e** The quadratic EO coefficient and half-wave voltage of PLDZT ceramics are analyzed as a function of Dy substitution level. Reproduced from ref. ^81^ with permission from Elsevier: Ceramics International

The chemical formula of lead lanthanum zirconate titanate, abbreviated as PLZT, is given by Pb_1-*x*_La_*x*_(Zr_*y*_Ti_1-*y*_)_(1-*x*/4)_O_3_. This represents the substitution of La^3+^ ions for a portion of the Pb^2+^ ions in PZT. Its ceramic powder can be synthesized through various methods, including solid-phase reaction^69^, sol-gel^70^, coprecipitation^71^, and hydrothermal processes^72^. The pioneering PLZT ceramics were fabricated by Haertling G.H. in 1970, utilizing ball milling and hot pressing sintering techniques^73^. The introduction of lanthanum doping significantly alters the ferroelectric and other properties of PLZT compared to PZT. The room temperature phase diagram of the system, depicted in Fig. 10a, reveals that as the lanthanum doping content increases, the AFE and ferroelectric four-direction regions expand. Notably, at a certain lanthanum content, the paraelectric phase emerges and expands, indicating a decrease in the Curie temperature (*T*_*c*_) with increasing La further divides the PLZT system’s phase diagram into three distinct regions based on the varying optical properties attributed to different PLZT compositions^74^. The memory region exhibits low coercivity, excellent polarization retention, and a high voltage constant. Conversely, the linear effect zone features a high coercivity and primary EO effect during saturation polarization, yielding a substantial EO coefficient. Uniquely to PLZT compared to PZT, the quadratic effect region showcases a secondary EO effect, which finds broad application in EO devices such as switches and modulators^75^. In 2012, B. Xia and colleagues published their investigation on PLZT-based ceramics, focusing on the fabrication and testing of transparent PLZT ceramics with La concentrations precisely tuned at 7.6, 7.8, and 8.0 mol%. Upon the application of an electric field of suitable intensity, these materials undergo a reversible phase transition from the AFE to the ferroelectric (FE) phase, accompanied by the intriguing phenomenon of light scattering. Upon removal of the electric field, the light scattering promptly dissipates, restoring the material’s transparency. This unique combination of high transmittance and low threshold properties in the absence of an electric field positions PLZT as a promising candidate for specialized optical modulators, including optical shutters and variable optical attenuators^76^.

PLZT ceramics exhibit remarkable EO properties, characterized by a substantial EO coefficient and high dielectric constant, which endow PLZT-based EO modulators with exceptional modulation capabilities, including low-voltage modulation and an extensive field of view. Nevertheless, the inherently low transmittance of PLZT crystalline ceramics poses a challenge, as it may result in a diminished modulation signal upon light’s traversal through the modulator and subsequent entry into the receiver. Consequently, the doping modification of PLZT_(*x*/70/30)_ ceramics with diverse element ions has emerged as a pivotal research focus for scholars globally^77^. In 2019, Zhu et al. published their research on the transmissivity of Al-doped PLZT_(8.0/69/31)_ ceramics^78^. Their study revealed that a minute quantity of Al doping enhances the spectral transmittance of PLZT_(8.0/69/31)_ ceramics, whereas a significant decrease in transmittance is observed with an increase in the Al doping concentration. Parallel to this, Sun et al. examined the impact of Er^3+^ and Yb^3+^ ion doping on the EO properties of PLZT ceramics, determining their EO coefficients to lie within the range of 0.38–0.53$\times {10}^{-16}{{\rm{m}}}^{2}/{{\rm{V}}}^{2}$, indicating a reduction in the EO coefficients upon the incorporation of rare earth ions^79^. Meanwhile, He et al. discovered that a modest amount of Dy doping enhances the polarizability, transmittance, and refractive index of PLZT_(12/40/60)_ ceramics, transforming these inherently linear EO materials into those exhibiting secondary EO effects, with EO coefficients reaching as high as 5.59$\times {10}^{-15}{{\rm{m}}}^{2}/{{\rm{V}}}^{2}$^80^. In 2014, Zeng et al. made a significant discovery, reporting that Dy-doped PLZT-based (PLDZT) EO device can exhibit remarkably high EO coefficients, reaching up to an impressive value of $4\times {10}^{17}{m}^{2}/{V}^{2}$ ^81^. Figure 10b depicts the transmittance spectra of PLDZT ceramics, varying with wavelength, for a fixed thickness of 0.35 mm. Notably, the transmittance values surpass 67% at 632.8 nm, experiencing a slight enhancement with increased Dy dopant concentrations. For an ideal transparent ceramic with thickness *t*, the theoretical transmittance *T* is formulated as *T* = (1 − *N*)^2^ e^(−*βt*)^ *N* = ((*n* − 1)/(*n* + 1))^2^, encompassing the refraction coefficient *N*, refractive index *n* of the sample, and the absorption-scattering factor *β*^82^. Specifically, PLZT(10/65/35) exhibits a refractive index of ~2.479, accompanied by 31% optical reflection losses at 632.8 nm due to double air/ceramic interface reflections, leading to a derived theoretical transmittance of 69%. However, with an appropriate antireflection coating, these losses can be mitigated, potentially yielding transparency nearing 97% of the theoretical value. The EO properties of PLDZT ceramics were assessed through birefringence Δ*n* measurements as a function of the electric field, utilizing 632.8 nm He–Ne laser light. Drawing from Haertling’s research^74^, Fig. 10c illustrates a typical setup for characterizing EO ceramics, where PLZT is positioned between crossed polarizers (P1 and P2), with the applied electric field oriented at 45° to the incident polarized light. As the voltage varies, so does the intensity of the detected emergent light, which achieves maximum intensity at half-wave retardation. This defines the half-wave voltage *V*_*λ/2*_^83^. The birefringence Δ*n* is calculated using *I/I*_*0*_ = sin^2^(π*lΔn/λ)*, where *I*_*0*_ and *I* represent incident and emergent light intensities, *l* is the ceramic thickness along the optical path, and *λ* is the wavelength^84^. The quadratic EO coefficient *R* can then be derived from *Δn* = −(1/2)*Rn*^3^*E*^2^ ^85^, with Fig. 10d illustrating the Δ*n*-*E* relationship, where *R* is proportional to the slope of the Δ*n* vs. *E*^2^ cure. Notably, Dy substitutions enhance *R* in PLZT (10/65/35), as evidenced by the data points for 0.02Dy, 0.04Dy, and 0.06Dy substitutions. At *V*_*λ/2*_, the calculated *R* values are 2.566 × 10^−^^16^(m/V)^2^, 2.83 × 10^−^^16^(m/V)^2^, 3.30 × 10^−^^16^(m/V)^2^, and 4.67 × 10^−^^16^(m/V)^2^, respectively. Similarly, the half-wave voltages decrease with Dy content, from 850 V to 610 V, 580 V, and 510 V. Figure 10e summarizes these findings, highlighting the positive correlation between Dy dopant content and quadratic EO coefficient, along with the inverse relationship with half-wave voltage. This underscores the intricate link between the EO and ferroelectric properties of PLZT materials^74^.

(1-*x*)Pb(Mg_1/3_Nb_2/3_)O_3_-*x*PbTiO_3_ (PMN-PT), is a superior relaxed ferroelectric material renowned for its exceptional ferroelectric, piezoelectric, and EO characteristics. The phase structure and, consequently, the material’s performance vary significantly with the proportion of PT. When PT content is below 0.31, PMN-PT assumes a trigonal phase, showcasing exceptional EO performance and high transparency, ideal for crafting EO switches and modulators with promising applications in optical communication. As PT content rises, PMN-PT undergoes a transition from trigonal to tetragonal phase, becoming suitable for high-temperature environments when PT exceeds 0.37. At the critical composition range between 0.31 and 0.37, PMN-PT resides at the morphotropic phase boundary (MPB), where both trigonal and tetragonal phases coexist, fostering remarkable ferroelectric, piezoelectric, dielectric, and electrostrictive properties^86^. Yin et al. discovered that the electric field sensor designed utilizing PMN-PT exhibits high performance, characterized by a quality factor nearing 10, a remarkable sensitivity reaching 4 pm, and the capability to measure electric field strengths as low as 20 mV^87^. Furthermore, electric field sensors crafted from EO materials boast the benefits of high-fidelity and broadband measurement capabilities, attributed to their all-dielectric design. Consequently, PMN-PT has found extensive application in the fabrication of electric field sensors.

The practicality of PMN-PT transparent ceramics as EO materials hinges not solely on their EO coefficient but is also influenced by parameters like light transmittance. Notably, the La-doped 0.75Pb(Mg_1/3_Nb_2/3_)O_3_–0.25PbTiO_3_, synthesized by Ruan et al. in 2010 through a two-stage sintering approach, boasted a secondary EO coefficient of 66 × 10^−16^ (m/V)^2^ and a transmittance of 65% at infrared wavelengths, marking the highest reported at that time^88^. More recently, in 2024, Hu et al. devised and successfully fabricated a highly transparent PMN-PT utilizing an economical two-step sintering process in an oxygen atmosphere, achieving a secondary EO coefficient of 42.1 × 10^−^^16^ (m/V)^2,89^. Furthermore, Badillo et al., in 2012, investigated the light transmittance of La-doped PMN-PT transparent ceramics within the compositional range of 0.11 ≤ *x* ≤ 0.15, exploring the correlation between properties like the secondary EO coefficient and the lead titanite content. Their findings revealed that at *x* = 0.14, the transmittance peaked, with 0.63 mm thick ceramics exhibiting transmittance of ~43% at 632 nm and 49% at 900 nm, while the secondary EO coefficient reached its maximum of 23.7 × 10^−^^16^ (m/V)^2^ at *x* = 0.13^90^. Fujii et al. (2017) successfully prepared PMN-PT ceramics using conventional sintering methods and measured their EO properties. They found that samples with *x* = 0, 0.1, and 0.2 had transmittances of 47%, 43%, and 53% at 1310 nm, respectively, and secondary EO coefficients of 6.3, 25.5, and 13.8 × 10^−^^16^ (m/V)^2^ ^91^.

**Fig. 11:**
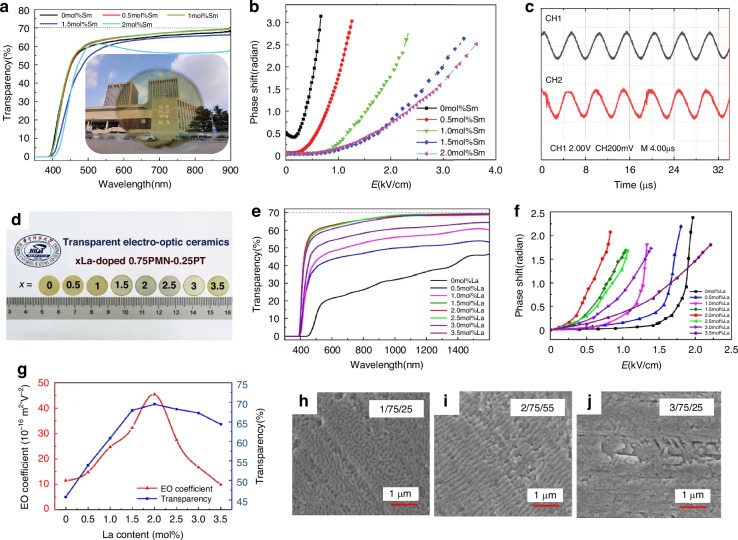
EO properties of PMN-PT material. **a** Transmittance spectra of Sm-doped PMN-PT ceramics, featuring a thickness of 0.85 mm. **b** The graphical representation of the phase shift varying with the applied electric field. **c** Modulation of sinusoidal signals occurring at a frequency of 200 kHz. R from ref. ^86^ with permission from John Wiley and Sons: Advanced Optical Materials. **d** Transparency analysis of 0–3.5 mol% La-doped 0.75PMN–0.25PT ceramics, accompanied by a photograph showcasing the transparency of PMN-PT ceramics at a thickness of 0.5 mm. **e** Optical transmission spectra of PMN-PT ceramics, exhibiting a thickness of 0.5 mm. **f** EO properties of 0–3.5 mol% La-doped 0.75PMN–0.25PT ceramics, specifically the phase shift plotted against the electric field. **g** The influence of La-content on both the EO coefficient and transparency. **h**–**j** Examination of domain morphologies for compositions of 1/75/25, 2/75/25, and 3/75/25, across multiple grain scales. Reproduced with permission from ref. ^95^

In 2021, Fang et al. embarked on a comprehensive study focusing on ultra-transparent PMN-PT ceramics. Their findings revealed that the incorporation of 0.5 mol% of Sm-doping into the PMN-PT significantly enhanced its transparency, achieving a remarkable level of up to 69.6%. Furthermore, this doped material exhibited an impressive quadratic EO coefficient, reaching as high as 35 × 10^−^^16^m^2^/V^2^ ^86^. Figure 11a vividly displays the transmittance spectra of Sm-doped PMN-PT ceramics, with a uniform thickness of 0.85 mm, across the wavelength spectrum from 300 to 900 nm. Notably, samples doped with 0 to 1.5 mol% Sm exhibit remarkable transparency exceeding 67% in the near-infrared region, peaking at 69.6% and 69.4% for 0.5 and 1 mol% doping, respectively, around 900 nm. This exceptional clarity is aptly illustrated in the inset, where the intricate details of buildings and a school motto are discernible through the ceramic. In line with previous studies, the transparency of PMN-PT ceramics escalates with wavelength, suggesting that at optical communication bands of 1310 and 1550 nm, the transparency of 0.5 and 1 mol% Sm-doped samples may surpass 70%^88,92,93^. Turning to Fig. 11b, a nonlinear relationship between phase shift (retardation) and electric field at 632 nm underscores the quadratic EO coefficient (Kerr effect) inherent in PMN-PT transparent ceramics, contrasting the linear EO behavior (Pockels effect) observed in PMN-PT single crystals^94^. As Sm content increases, the electric field required to attain a specific phase shift, such as 2.5 radians, escalates systematically. Despite their outstanding EO properties, the challenge of crafting highly transparent PMN-PT ceramics has hindered their exploration in optical communication applications. Ze Fang’s team has boldly ventured into this territory by designing an EO modulator leveraging 0.5 mol% Sm-doped PMN-PT transparent ceramic. Based on the modulator’s signal transmission setup, integrating a ① laser, ② polarizer, ③ transparent ceramic, ④ analyzer, and ⑤ silicon photocell, ⑥ signal generator, and ⑦ oscilloscope, complemented by LabVIEW interfaces or audio outputs. Figure 11c showcases the modulator’s performance, effectively modulating sinusoidal signals at 200 kHz with minimal distortion. The original electric signal (CH1, black) is faithfully reproduced in the light intensity signal (CH2, red) captured by the silicon photodetector. By optimizing the ceramics’ dimensions, driving voltage can be reduced while modulation frequencies are enhanced, further underlining the potential of this innovative modulator in high-frequency optical communication systems^86^. Notably, the EO properties of PMN-PT ceramics were optimized when the lead titanate content was approximately *x* = 0.1 and 0.25, with further enhancement observed under La doping. In 2024, Hu et al. discovered that doping samples with 2 mol% La facilitated the parallel stacking of polar nanodomain structures, enabling faster and easier polarization switching, as shown in Fig. 11h~j^95^. This ordered distribution of PNRs was crucial for inducing significant EO effects, transparency, and piezoelectric response, resulting in a record-high transparency of 69% in the near-infrared (IR) wavelength (1550 nm) alongside an exceptionally high secondary EO coefficient of 45.4 × 10^−^^16^(m/V)^2^. This achievement effectively balances the transparency and the EO coefficient. The relationship between its phase and the electric field is depicted in Fig. 11f, while the relationship between the EO coefficient and concentration is illustrated in Fig. 11g. As research progresses, the sintering process, doping substances, and their concentrations in PMN-PT will continue to be the focus of the investigation. Notably, a free spectral range (FSR) of 1.34 nm is shifted by an electric field <20 V, achieved with a single cavity thickness of 350 μm. This translates to a wavelength shift of ~1.34 nm per 0.1 V/μm of electric field, significantly lower than previous reports by Chen et al.^96^, reaffirming the substantial EO effect exhibited by PMN-PT ceramics^95^.

Given the heat generated by high-power lasers when PMN-PT ceramics are employed, it is imperative to investigate the influence of temperature on their EO properties. In 2017, Zhang et al. conducted an experiment examining the EO response of PMN-PT transparent ceramics across temperatures ranging from 275 K to 325 K, elucidating the temperature’s mechanism on the EO effect from a ferroelectric perspective. Their findings reveal a gradual decrease in the EO coefficient of PMN-PT ceramics with rising temperatures, with values diminishing from 6.6 × 10^−^^15^(m/V)^2^ at 276.2 K to 2.5 × 10^−^^15^ (m/V)^2^ at ~323 K^97^. Consequently, to ensure optimal EO performance, temperature stability is crucial during the utilization of PMN-PT ceramics.

#### EO single crystals

**Fig. 12:**
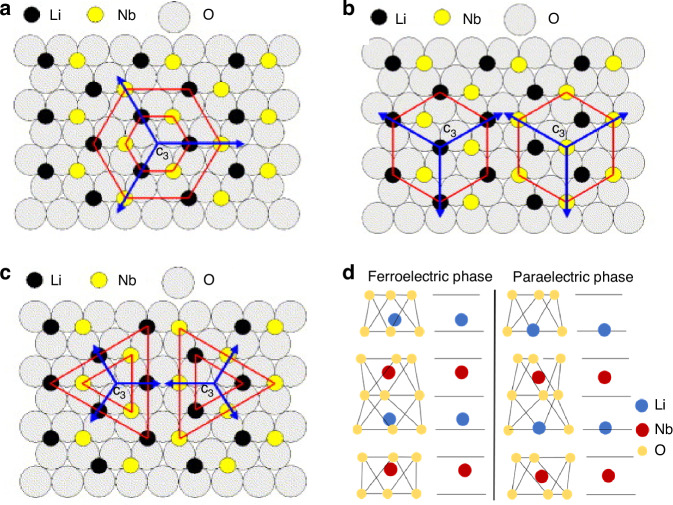
Crystal structure of LNO system. **a**, **b** Hexagonal domains structure in SLN, **c** triangular domains structure in CLN Reproduced from ref. ^98^ with permission from Elsevier: Materials Science and Engineering B. **d** Crystal structure of lithium niobate in ferroelectric and paraelectric phase

LNO is a highly valuable optical material with excellent electro-optic, optical nonlinearity, acousto-optic, ferroelectric, and piezoelectric properties, as well as a broad optical transparency window and a high refractive index. The crystal lattice of LNO belongs to the 3 *m* point group type, with trigonal rotational symmetry around the c-axis (also known as the Z-axis). Typically, LNO unit cell exhibits mirror symmetry along the YZ plane, which gives rise to two distinct microscopic domain structures: the hexagonal domain structure found in near-stoichiometric lithium niobate (SLN), and the triangular domain structure observed in congruent lithium niobate (CLN), as depicted in Fig. 12a~c. Notably, SLN boasts a lower count of inherent defects compared to CLN crystals^98^, contributing to its unique properties. Under a strong external electric field applied along the +c direction, Li and Nb atoms drift towards the -c direction, with Li atoms crossing the oxygen plane to reach the other side, causing a reversal in both the spontaneous polarization field direction and the domain orientation within the lattice. This characteristic allows for periodically poling LNO domains to achieve quasi-phase matching, which is of significant importance in nonlinear optics.

LNO has an optical transparency range between 350 nm and 5 μm, covering the visible, near-infrared, and a portion of the mid-infrared spectra. It exhibits birefringence and a relatively high refractive index (at a wavelength of 1550 nm, *n*_*o*_ ≈ 2.211*,n*_*e*_ ≈ 2.137), allowing it to be used on common low-refractive-index substrates (such as silicon dioxide and sapphire) for constructing waveguide structures. Unlike materials such as silicon and silicon nitride, LNO is a crystal with non-centrosymmetric structure, exhibiting strong linear EO effects and a high second-order nonlinear coefficient (*d*_*33*_ = 31 pm/V)^99^. The linear EO effect of LNO can be utilized to construct EO modulators for loading information onto the optical domain, while its high second-order nonlinear coefficient and ferroelectric properties allow for the construction of efficient frequency conversion devices through periodic poling reversal of its ferroelectric domains, achieving effects such as optical second-harmonic generation, sum-frequency generation, difference-frequency generation, and parametric down-conversion. Additionally, LNO possesses a high third-order nonlinear coefficient (*n*_*2*_ = 1.8 × 10–19 m^2^/W), making it suitable for generating four-wave mixing and Kerr combs^11^. Figure 12d^100^ vividly illustrates the positions of Li and Nb atoms in both the paraelectric and ferroelectric phases.

**Fig. 13:**
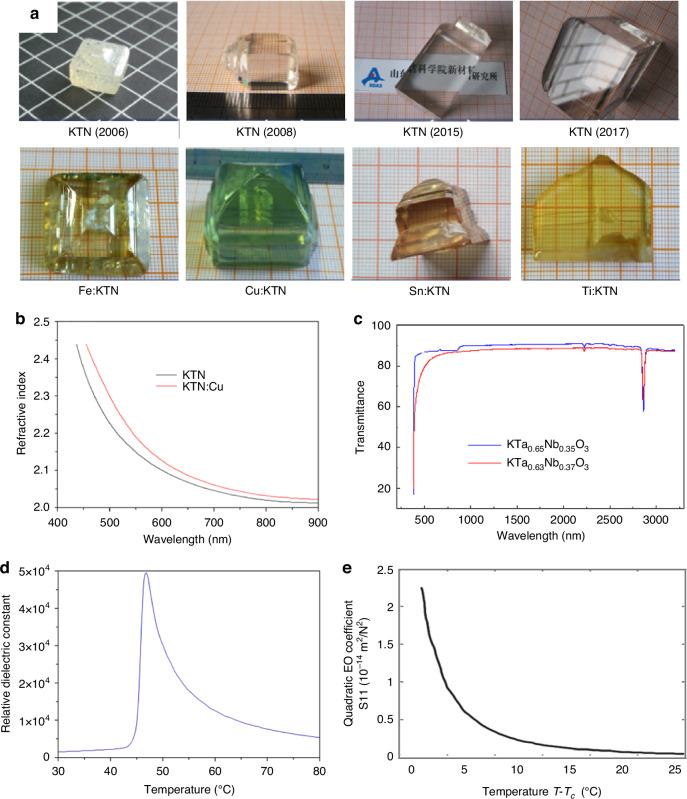
Various aspects of as-grown KTN crystals with diverse compositions, sizes, and doped ions at the device level. Reproduced from ref. ^106^ with permission from Elsevier: Journal of Materiomics. **a** Device levels of the as-grown KTN crystals with different compositions, different sizes, and different doped ions. **b** The linear optical properties of the KTN crystal series, specifically the refractive index dispersion curves for both KTa_0.63_Nb_0.37_O_3_ and Cu-doped KTa_0.63_Nb_0.37_O_3_ single crystals. **c** The transmittance spectra of the same crystals. **d** The dielectric properties, presenting the relative dielectric constant of KTa_0.65_Nb_0.35_O_3_ as measured by Yagi et al. from NTT Japan. **e** The temperature-dependent Kerr coefficient *S*_*11*_, highlighting its temperature dependence in KTa_0.65_Nb_0.35_O_3_

Despite LNO, KTa_1-*x*_Nb_1-*x*_O_3_ (KTN) crystals exhibit remarkable EO properties, such as a substantial secondary EO coefficient^101^, wideband refraction^102^, and non-diffractive light wave propagation^103^. These crystals possess a multi-faceted periodic structure, encompassing orientations at 45°, 90°, 135°, and 180°, as well as within the *x-y* and *y-z* planes. KTN crystals embody continuous solid solutions with tunable Ta/Nb ratios^104^. Concurrently, the *T*_*c*_ undergoes variation in accordance with the Ta/Nb ratio^105^, facilitating phase transitions. At around 300 K, the material transitions from a ferroelectric orthogonal phase to a tetragonal phase at a ratio of 0.5, aligning with *T*_*c*_. At 400 K, it shifts from the tetragonal to a paraelectric cubic phase. This results in a composite ferroelectric domain at room temperature, with an optimal Ta/Nb ratio and a three-dimensional distributed polarization direction that inherits cubic symmetry. According to Bragg diffraction theory, the wall of a 90° domain in a ferroelectric crystal aligns at 45° to the crystal’s major axis, whereas the domain wall of a 180° domain can be either parallel or perpendicular to it. It is essential for both 90° and 180° domains to coexist within ferroelectric supercells for KTN crystals to exhibit a six-directional periodic ferroelectric domain structure simultaneously within the crystal plane. These ferroelectric microstructures induce periodic fluctuations in the refractive index, enabling linear light diffraction. As depicted in Fig. 13a, both the pristine and ion-doped KTN crystals exhibit a cubic or quadrilateral columnar morphology^106^, aligning with the growth traits characteristic of cubic-phase crystals. X-ray diffraction orientation analyses confirm that the predominant crystal plane family exposed is the cubic monomorphic $\left\{100\right\}$, with a minority of crystals also displaying $\left\{110\right\}$ facets, without any additional monomorphic structures emerging^105^. Notably, the KTN crystal attained dimensions of 35 mm × 37 mm × 58 mm, positioning it among the largest reported to date^107,108^. Compositional variations in Ta or Nb within the grown KTN crystal were assessed to be within the order of 10^−5^ mm^−1^ ^109^, fulfilling the standards for laser modulation device applications. The refractive indices of the KTN crystal series escalated with increasing Nb concentration, ranging from 2.1520 and 2.2256 for KTaO_3_ to 2.1877 and 2.27170 for KTa_0.63_Nb_0.37_O_3_ at 1539 nm and 633 nm, respectively^110^. Figure 13b illustrates the refractive index dispersion profiles of KTa_0.63_Nb_0.37_O_3_ and Cu:KTa_0.63_Nb_0.37_O_3_ single crystals, with the latter demonstrating superior reflectivity across the 400–900 nm wavelength spectrum, attributed to the residual Cu^2+^ ions, which enhanced the refractive index due to their mobility and high electronic polarizability^111,112^. Absorption spectroscopy reveals that both KTN and *M*:KTN (*M* = Fe, Cu, Sn, and Ti) crystals exhibit high transmittance in the near-ultraviolet, visible, and near-infrared regions (Fig. 13c), favorable for optical applications. In 2009, Yagi et al. reported a remarkable relative dielectric constant of 50,000 for KTa_0.65_Nb_0.35_O_3_ at a transition temperature of 47 °C (Fig. 13d), ranking among the highest reported for any known material^113^. Additionally, Fig. 13e depicts results and calculation methods of the Kerr coefficient, *S*_*11*_, of KTN at varying temperatures relative to the Curie point, indicating that proximity to the Curie point yields higher *S*_*11*_ values, translating to lower half-wave voltages for EO applications. Assuming longitudinal laser modulation with a field perpendicular to the light path, the half-wave voltage for the linear EO effect can be formulated as V_π_ = *d*/*n*^3^*rL*, where *r* is the effective linear EO coefficient of paraelectric KTN, and *d*, *n*, and *L* represent the electrode gap, refractive index, and crystal length, respectively. The maximum *S*_*11*_ value of 2.2 × 10^−14^ m^2^/V^2^ corresponds to an effective linear EO coefficient of 2.2 × 10^−9^ m/V, approximately tenfold higher than typical Pockels cells, emphasizing the superior efficiency of KTN crystals over conventional linear EO materials^106^.

### Typical EO thin films

#### LNO thin films

LNO possesses a high third-order nonlinear coefficient (*n*_*2*_ = 1.8 × 10–19 m^2^/V), making it suitable for generating four-wave mixing and Kerr combs. Single-crystal LNO thin films on insulators (LNOI or SOI) hold significant promise as platforms for integrated optics due to their high refractive index contrast, which enables strong light guiding. These LNO thin films retain the superior optical properties of bulk LNO material. When combined with the exceptional nonlinear, acousto-optic, and EO characteristics of LNO, LNOI supports the development of high-performance, compact photonic devices. Notable examples include EO modulators, nonlinear wavelength converters, and microresonators. This can be attributed to its material properties.

Deposition methods such as evaporation, sputtering, and chemical vapor deposition typically result in polycrystalline LNO thin films. These polycrystalline films often cause high optical transmission loss due to light scattering at grain boundaries. In contrast, single-crystal LNO thin films can be physically extracted from bulk LNO material and transferred to a supporting substrate through processes like ion implantation, direct bonding, and thermal annealing. To support the LNO thin film, various handle materials have been developed, including LNO itself, sapphire, SiC, and quartz. Currently, the maximum size for LNOI wafers is 8 inches. The lapping and polishing method can also produce high-quality LNOIs with minimal impact on crystal quality but imposes stringent requirements on thickness uniformity control.

**Fig. 14:**
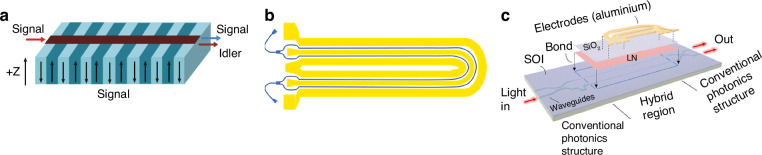
Illustrations of the LNO-based EO modulators. **a** Schematic of the periodically poled lithium niobate (PPLN) waveguide. Reproduced with permission from ref. ^116^. **b** Schematic illustration of the typical folded Mach-Zehnder interferometer modulator. **c** Mach-Zehnder modulator reported by Weigel et al. Reproduced from ref. ^119^ with permission from Optica Publishing Group

Recently, advancements in ferroelectric domain engineering of lithium niobate have transcended from one-dimensional to two- and three-dimensional realms^114^, with periodically polarized lithium niobate (PPLN) emerging as a vital component in frequency conversion and nonlinear beam shaping applications^115^. Given LNO’s polarization exclusivity along the *z*-axis, PPLN optimizes performance for light polarized parallel to this axis, maximizing the utilization of the high *r*_*33*_ coefficient (Fig. 14a). Positioning the waveguide on the PPLN wafer’s surface confines the pump beam throughout the entire interaction length, drastically enhancing conversion efficiency^116^. Over the past quarter-century, the realm of integrated lithium niobate photonics has been firmly anchored on the foundation of high-quality lithium niobate-on-insulator (LNOI) technology^117^, complemented by cutting-edge photonic integrated circuit advancements for the precise etching of nanophotonic waveguides and micro photonic modulators. Despite these achievements, thin-film lithium niobate (TFLN) continues to hold immense promise for future development, fueled by its inherent advantages of low driving voltage, exceptional EO properties, high speed, and compact size.

The modulation bandwidth of EO modulators is inherently constrained by the EO coefficient of the utilized materials. Advancements in waveguide processing technology have paved the way for the development and implementation of TFLN, effectively addressing the shortcomings of traditional bulk modulators. TFLN boasts a waveguide refractive index difference exceeding 0.7, significantly enhancing the waveguide’s light confinement capabilities. This not only results in a reduction in modulator size but also lowers the modulation voltage^61^. Building upon this progress, in 2021, Hu et al. ^118^ showcased a folded Mach-Zehnder interferometer modulator based on X-cut LNO thin films, accompanied by an efficient polarization program for device activation. The device’s innovative structure is schematically illustrated in Fig. 14b. The proposed modulator design innovatively achieves a shorter device length without compromising its performance. Specifically, the fully polarized folded modulators, upon fabrication and measurement, exhibit a *V*_*πL*_ of ~2.74 V cm, alongside a remarkable 3 dB EO bandwidth of roughly 55 GHz^118^. Notably, in a 2018 study, Peter O. Weigel et al. introduced the first Mach-Zehnder EO modulator leveraging an unpatterned, unetched TFLN layer integrated with a single-mode silicon photonic circuit. This innovative design achieved electrical modulation bandwidths surpassing 100 GHz, as illustrated in Fig. 14c^119^. Furthermore, the manufacturing process, which eschews LNO etching or sawing and aligns with standard silicon photonics foundry protocols, fosters compatibility between lithium niobate and silicon photonics. This underscores the potential of TFLN materials to revolutionize the manufacturing of EO modulators, enabling the creation of more compact and potent devices. Consequently, the integration of TFLN materials paves the way for advancements in fiber-optic communication technology, fostering its continued growth and development.

#### BTO thin films

**Fig. 15:**
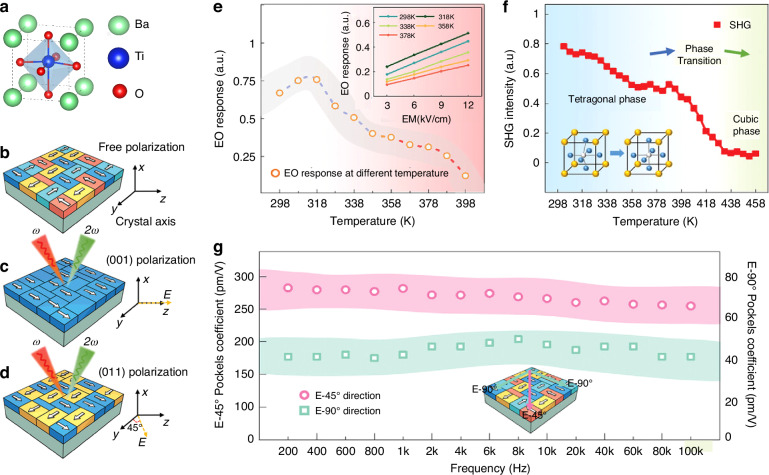
Domains and EO properties of BTO thin film. **a** Schematic illustration of the BTO structure. **b** The domain variants in the BTO thin film exhibit free polarization when no electric field is applied. **c** Evolution of domain variants in the BTO thin film when an electric field is applied along the *z* direction. **d** Evolution of domain variants in the BTO thin film when an electric field is applied along the *yz*-direction. **e** Temperature dependence of the EO response in BTO thin films, with the inset showing the linear relationship of the EO response with the modulation electric field at different temperatures. **f** Temperature dependence of the SHG intensity, revealing the phase transition process of the BTO thin films. **g** EO coefficients of BTO materials at different electric field angles and frequencies Reproduced from ref. ^143^ with permission from John Wiley and Sons:: Advanced Optical Materials

Recently, BTO’s exceptional EO performance has garnered significant attention. The traditional commercial LNO wave-conductive optical modulator, with an EO coefficient of ~31 pm/V, faces limitations in surpassing the 40 GHz single-channel bandwidth barrier. However, the escalating demands of the optical market, particularly for high- and ultra-high-bandwidth optical modulators, necessitate advancements beyond this threshold, with 40 GHz considered the minimum and 100 GHz the core bandwidth. Contrastingly, BTO boasts an EO coefficient that significantly surpasses that of LNO. BTO, a classic perovskite ferroelectric, is shown in Fig. 15a with a melting point of 1618 °C and Curie temperature of 130 °C, experiencing intricate crystal phase transformations upon cooling^120^. This progression includes hexagonal, cubic, tetragonal, orthorhombic, and triclinic phases with decreasing crystal symmetry. BTO excels in lead-free ferroelectric materials, essential for electronic ceramics and diverse applications like MLCCs, PTC thermistors, optoelectronics, and RAMs^121–123^.

In 2005, Sun et al. demonstrated the potential of BTO-based EO modulators by reporting an ideal BTO film on MgO substrates with an impressive *r*_*51*_ value of 730 pm/V^124^. Building upon this, in 2018, Abel et al. achieved a milestone by integrating micro-nanostructured BTO on silicon and extracting an EO coefficient of up to 923 pm/V, a staggering 30-fold increase over LNO’s maximum coefficient and the highest value recorded for BTO materials to date^8^. Moreover, in 2015, Sun et al. explored the feasibility of 100 GHz bandwidth EO modulators utilizing c-axis grown BTO crystal thin films. Their research underscored that under optimal conditions, including an extremely high EO coefficient *r*_*51*_, a tailored nonlinear EO modulation model, and an optimized device structure or waveguide/electrode dimensions, MZI-structured EO intensity modulators featuring c-axis growth BTO crystal thin-film waveguides can attain ultra-high bandwidths exceeding 100 GHz^125^. Furthermore, BTO materials hold the advantage of seamless integration with existing CMOS manufacturing infrastructure, thereby mitigating costs, simplifying production processes, and enhancing device yields for BTO-based devices. Recently, in 2023, Dong et al. showcased a groundbreaking monolithic BTO MZI modulator, leveraging a low-loss all-BTO waveguide. This modulator achieved a V_πL_ of 2.32 V cm, which is on par with the cutting-edge LNO EO modulators, and its effective linear EO coefficient (*r*_*eff*_) was calculated to be 89 pm/V^126^. These remarkable findings underscore the practicality of CMOS-compatible monolithic EO device architectures, realized through the epitaxial sputtering of BTO onto silicon substrates.

The EO properties of BTO films are influenced by various factors, including domain structure, film thickness, substrate material, and the preparation process. Numerous recent studies have underscored the intimate link between the EO characteristics of BTO and its domain structure, as evidenced in literature^8,127–135^. Furthermore, research into domain structure manipulation in BTO has been ongoing since the dawn of the 21st century^136–141^, yet a significant breakthrough in enhancing the EO coefficient has only emerged recently. Li et al. achieved this milestone by demonstrating that the high-quality epitaxial growth of a GdScO_3_ buffer layer effectively mitigates substrate strain, thereby boosting the proportion of switchable domains. This enhancement enabled the GdScO_3_-buffered BaTiO_3_ film to exhibit a notably wider multi-stage operation phase shift window of 182% and a superior effective linear EO coefficient of 175 pm/V, marking a pivotal advancement in EO coefficient control^142^.

Additionally, Wen et al. observed that antiparallel domains in multi-domain BTO films do not contribute to the net EO response^143^. By increasing the DC bias, they were able to align more antiparallel domains, subsequently augmenting the EO response. As shown in Fig. 15b, in the absence of an electric field, BTO thin film exhibits four equivalent polarization directions due to 180° phase differences in non-parallel domains. Applying an electric field of 60 kV cm^−^^1^ along the *z* direction realigns the domains (Fig. 15b), leading to a single-domain structure. Adjusting the electric field direction to 45° results in a twofold symmetry due to the coherent superposition of in-plane 90° domains, and a significant EO response due to the contribution of *r*_*42*_ (Fig. 15c). The analysis of BTO films reveals that the temperature and frequency dependence of the EO effect is significantly influenced by domain dynamics (Fig. 15d, e). As temperature increases, BTO transitions from the orthorhombic to the tetragonal phase, enhancing spontaneous polarization and affecting the contributions of *r*_*42*_ and *r*_*51*_ coefficients induced by in-plane domains. The EO response initially increases with temperature due to thermal effects but weakens as depolarization occurs, aligning with the spontaneous polarization change trend in BTO bulk. The Curie temperature of multi-domain BTO thin films is estimated to be around 428 K, which is higher than the bulk due to factors like interfacial stress (Fig. 15g). The EO effect in BTO films shows frequency dependence, with domain switching playing a role. However, the modulation bandwidth in BTO thin film greatly exceeds the dynamic EO test limit, suggesting that frequency dependence is not a major limiting factor for BTO thin film applications.

#### PZT thin films

**Fig. 16:**
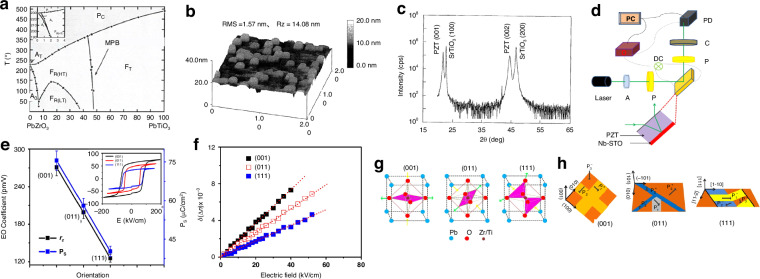
Phase and EO properties of PZT material. **a** Phase diagram of PbZrO_3_-PbTiO_3_ system. Reproduced from ref. ^145^ with permission from Taylor & Francis Group. **b** AFM of 270-nm-thick epitaxial PZT thin film crystallized on SrTiO_3_(100) at 650 °C Reproduced from ref. ^148^ with permission from IOP science: Japanese Journal of Applied Physics. **c** XRD pattern of PZT thin film waveguide grown on SrTiO_3_ (1100) substrate. Reproduced from ref. ^150^ with permission from Springer: MRS Online Proceedings Library. **d** The experimental setup designed for reflection-based measurement of EO coefficients comprises key components: an Attenuator (A) to adjust light intensity, a Polarizer (P) to select polarization, a Compensator (C) for fine polarization adjustment, a Photodetector (PD) converting light to electrical signals, an Oscilloscope (O) for signal visualization, a Personal Computer (PC) for data acquisition and analysis, and a Direct Current (DC) source from an amplifier to apply the external electrical field. **e** An investigation delves into the correlation between the linear EO coefficient and spontaneous polarization in PZT thin films with (001)-, (011)-, and (111)-orientations. The inset provides polarization-electric field hysteresis loops, illustrating the field-induced polarization dynamics. **f** The birefringence, denoted as *δ*(Δ*n*), is plotted against the external electrical field (*E*) for all three PZT thin films, elucidating their EO behavior. **g** Schematic representations illustrate the effect of strain on the oxygen octahedra configuration within the system for PZT thin films of various orientations: (001)-, (011)-, and (111)-oriented. **h** Schematic diagrams showcase the domain structures in PZT thin films of different orientations: (001)-oriented films exhibit a predominant P_3_^+^ (orange) phase alongside minor P_1_^+^ and P_2_^−^ (yellow) domains; (011)-oriented films feature a major P_3_^+^ (orange) phase, accompanied by minor P_2_^+^ and P_2_^−^ domains (blue), and trace amounts of P_1_^−^ (gray); whereas (111)-oriented films display intricate nanotwinned domains comprising P_1_^−^ (yellow), P_2_^−^ (blue), and P_3_^−^ (orange). Reproduced from ref. ^151^ with permission from AIP Publishing

In comparison to BTO, PZT materials featuring the perovskite structure exhibit superior piezoelectric properties, positioning them as one of the most extensively researched and utilized piezoelectric materials. PZT, with the chemical formula PbZr_*x*_Ti_1-*x*_O_3_, is a continuous solid solution that blends AFE materials: lead titanate (PbTiO_3_) and lead zirconate (PbZrO_3_). Figure 16a depicts the binary phase diagram of the PZT system, where P_C_ signifies the paraelectric cubic phase, a state where the temperature surpasses PZT’s Curie temperature, causing it to lose its ferroelectric and piezoelectric characteristics. F_R_ and F_T_ represent the ferroelectric triangular and tetragonal phases of PZT, respectively. The “Zr”-rich components adopt a rhombohedral structure (Zr:Ti > 54:46), while the “Ti”-rich components exhibit a tetragonal structure (Zr:Ti < 48:52). The ferroelectric and paraelectric phases of PZT are delineated by *T*_*c*_ lines, revealing an increase in PZT’s Curie temperature with a higher PbTiO_3_ content. Additionally, A_T_ and A_0_ signify AFE and AFE orthogonal phases, respectively^144^. At temperatures below the Curie point and around a 53/47 Zr/Ti ratio, a zone of coexistence between the triangular and four-direction phases emerges, known as the quasi-homomorphic phase boundary (MPB). PZT ceramics composed near the MPB exhibit optimal piezoelectric and dielectric properties, with electrical characteristics largely immune to temperature fluctuations, ensuring excellent temperature stability^145,146^. However, the presence of lead oxide as a constituent in PZT poses significant toxicity concerns, particularly exacerbated by its volatility at elevated temperatures, leading to environmental pollution during calcination and sintering processes. Consequently, recent years have witnessed numerous regulations banning or restricting the use of lead (Pb)-containing materials, propelling the pursuit of lead-free piezoelectric alternatives. Nevertheless, PZT continues to be the preferred material until lead-free substitutes with comparable or superior piezoelectric properties are discovered^147^.

In the realm of integrated optics, numerous applications rely heavily on the deployment of thin-film planar waveguides. Notably, PZT boasts not just the capability to form intricate optical waveguide structures but also exceptional properties like a high dielectric constant and an elevated EO coefficient. For the fabrication of PZT-based optical waveguide devices, heteroepitaxy emerges as a pivotal technique, given the absence of monocrystalline PZT wafers with small surface roughness (Fig. 16b)^148^. Heteroepitaxy enables the realization of a vast selection of ferroelectric thin film and substrate material combinations, encompassing integration with semiconductor devices that utilize MgO on GaAs structures^149^ or sapphire R-plane substrates for SOl applications^148^, as well as the development of low-voltage optical waveguide devices situated on conductive substrates^150^. Figure 16c exemplifies this, showcasing the XRD pattern of a 90 nm-thick epitaxial PZT film, successfully crystallized on a SrTiO_3_ (100) substrate at 650 °C. The refined ellipsometric EO measurement setup, featuring a longitudinal geometry, is depicted in Fig. 16d. Akin to the reflection geometry, an incident laser beam is focused onto the top electrode of the PZT thin film at a 45° angle. Upon application of an electric field, the laser beam reflects and traverses back through the film. Subsequently, an oscilloscope translates the resulting phase shift into an intensity modulation, which is then quantified by a photodetector synchronized to the excitation voltage frequency. Figure 16e concisely summarizes the birefringence variations observed in PZT thin films of varying growth orientations as a function of the applied electric field (*E*)^151^. Fig. 16f highlights the correlation between the linear EO coefficient and spontaneous polarization, revealing symmetric and well-saturated polarization-electric field hysteresis loops for all PZT films, regardless of their orientation (inset). Notably, the spontaneous polarizations of the samples are 75.5, 58.0, and 40.2 μC/cm² respectively. Figure 16g provides a schematic representation of the in-plane strain dynamics, with the underlying Nb-STO substrate (green) and PZT thin film (yellow) depicted.

It is noteworthy that PZT films near the morphotropic phase boundary exhibit a tetragonal structure with lattice parameters a = 4.036 and c = 4.146^152^, whereas Nb-STO possesses a cubic phase with a = 3.905. As illustrated in Fig. 16h, P_1_^+/−^, P_2_^+/−^, and P_3_^+/−^ denotes domains with polarizations aligned along the positive and negative (100), (010), and (001) axes, respectively. It has been established that (001)- and (011)-oriented PZT films exhibit a typical polydomain structure, dominated by P_3_^+/−^ domains and complemented by minor P_1_^+/−^ or P_2_^+/−^ domains. Conversely, (111)-oriented PZT films display a complex, metastable domain pattern characterized by a high density of nanotwinned domains, leading to a degeneration of polarization variants^151,153^. Ban et al. (2022) reported on PZT films grown via chemical solution deposition, exhibiting an EO coefficient of ~133 pm/V near the MPB, alongside negligible optical absorption losses across the 600 to 2500 nm wavelength range. Furthermore, they demonstrated a PZT MZI modulator on a SiO2/Si substrate, achieving a V_πL_ of 1.4 V·cm, a transmission loss of 1.8 dB/cm, and a 3 dB bandwidth of 12 GHz^154^. Zhu et al. delved into the impact of transparent substrates on the EO properties of lead zirconate titanate films. Their study revealed linear EO coefficients of 219.6 pm/V, 28.5 pm/V, and 69.9 pm/V, respectively, for PZT films near the MPB grown via RF sputtering on three distinct substrates: Corning 1737 aluminosilicate glass, indium tin oxide, and MgO single crystals^155^. These findings underscore the significance of substrate selection in achieving highly crystalline PZT films with minimal stress and defects, as the linear EO coefficient varies significantly depending on the substrate.

#### PLZT thin films

**Fig. 17:**
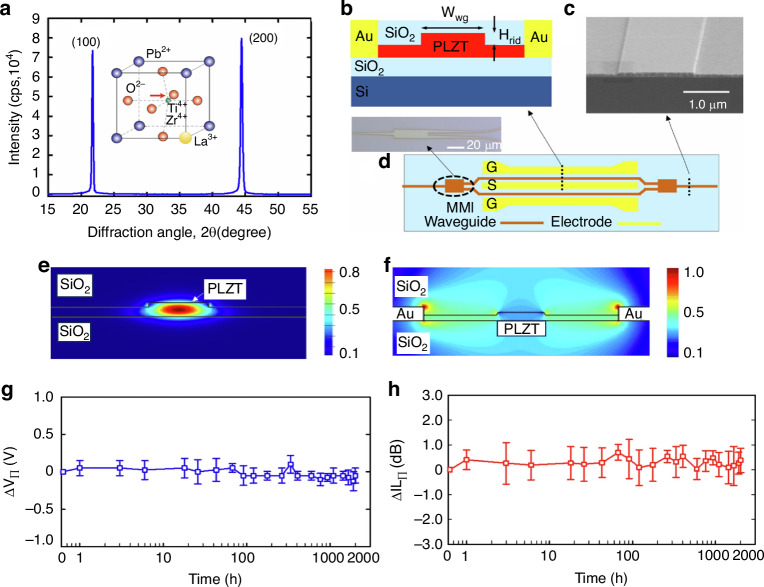
PLZT film and modulator reproduced with permission from ref. ^156^. **a** XRD pattern for a 200 nm-thick PLZT film prepared on a SiO_2_/Si substrate whose surface was coated with an intermediate layer. **b** Schematic showing the cross-section of the phase shifter part of the modulator. **c** Angled scanning electron microscopy image of the PLZT waveguide. **d** Schematic overhead view of a PLZT modular chip. Inset: magnified optical image of the MMI section. **e** Simulation of the optical electric field distribution along a cross-section of the PLZT waveguide. **f** Simulated RF electric field distribution. **g**, **h** *V*_π_ and optical insertion loss data, respectively, acquired from the PLZT modulator at 85 °C for 2000 h. Error bars indicate multiple sample variations

PLZT films enjoy extensive application in the electro-optics sector, where lanthanides play a pivotal role as intermediate layers during their fabrication. Notably, La_2_O_2_CO_3_ exhibits a distinct out-of-plane tetragonal orientation along the c-axis, characterized by lattice parameters a = 4.07 Å and c = 13.49 Å^35^, which is highly advantageous when employed as an intermediate layer atop SiO_2_ to yield c-axis-oriented PLZT films. The fabrication process entails iterative spin coating and heating cycles, culminating in annealing the film at 600 °C under O_2_ flow to promote the formation of crystalline PLZT^156^. Subsequently, the PLZT films were thoroughly analyzed via X-ray diffraction (XRD) utilizing *θ*–2*θ* scanning techniques, with a typical diffraction pattern presented in Fig. 17a. Here, the measured 2*θ* represents the diffraction angle in relation to the incident beam, where θ corresponds to the Bragg angle of the crystal. Distinct, intense peaks observed at 2θ = 21.95° and 44.72° correspond to the (100) and (200) crystallographic planes, respectively, indicative of ferroelectric domains with a square or rhombic lattice structure. This favorable outcome stems from the excellent lattice compatibility between PLZT (with a lattice constant a = 4.05 Å) and La_2_O_2_CO_3_, coupled with optimal environmental conditions such as appropriate O_2_ flow rates and substrate temperatures^156^.

The deposited PLZT film demonstrates a random in-plane dipole orientation relative to the surface normal, precluding the exhibition of EO effects without polarization. Polarization is crucial for inducing the ferroelectric dipole properties necessary for EO modulation applications. To achieve this, metal electrodes are deposited onto PLZT waveguides, facilitating both polarization and modulation. Figure 17b illustrates the cross-sectional schematic of the PLZT modulator, highlighting its waveguide design that maximizes optical confinement for efficient EO modulation. This is accomplished by fashioning a ridged waveguide on a 300 nm-thick PLZT film, with ridge dimensions of 1.5 μm width (*W*_*wg*_) and 100 nm height (*H*_*rid*_), as visible in Fig. 17c via scanning electron microscopy. The MZI unit, depicted in Fig. 17d, utilizes a 1 × 2 multimode interference coupler to split the incident laser beam into two waveguides, leading to a 2.5 mm long phase shifter. The electrodes adopt a coplanar ground-signal-ground configuration, enabling the electrical signal application to each phase shifter for push-pull MZI operation. In the phase shifter, the electric field is oriented along the surface plane, tailored for transverse electromagnetic (TE) mode operation, as evidenced by the calculated electric field distribution for the TE0 mode in Fig. 17e. Due to PLZT’s higher refractive index compared to SiO_2_, the upper and lower cladding confine the longitudinal modal propagation, concentrating the optical mode within the PLZT layer, achieving an estimated integration factor of 73%. Figure 17g shows a plot of the change in *V*_π_ over time, as obtained from a trial in which the device was heated in the air with measurements recorded at specific time intervals. The results confirm that the static EO effect for this PLZT modulator remained consistent at 85 °C for a time span of 2000 h. In addition, Fig. 17h establishes that the insertion loss showed no variation when the temperature was varied. Notably, testing at 85 °C was important to ensure the industrial-grade reliability necessary for optoelectronic devices^156^.

## Emergent binary EO materials

HfO_2_, renowned for its ease in forming a multiphase structure and its superior iron polarization capability in comparison to BTO^157–162^, coupled with its exceptional silicon compatibility, holds immense promise as a novel gate material poised to replace SiO_2_, thereby unveiling vast research opportunities. Similarly, Mg-doped ZnO and Sc-doped AlN, being emergent binary ferroelectric materials with relative higher remnant polarization above 100 μC/cm^2^ compared with conventional perovskite ferroelectrics^161,163^. They exhibit promising potential for EO modulation applications, owing to their CMOS compatibility and inherently low dielectric constant.

### HfO_2_-based ferroelectrics and EO effect

Recently, studies have unequivocally demonstrated that orthorhombic *Pbc*2_1_^164^ and rhombohedral *R*3*m*^165^ phases within HfO_2_-ZrO_2_-based thin films exhibit notable ferroelectric properties. This breakthrough holds immense practical potential, as both HfO_2_ and ZrO_2_ possess exceptional silicon compatibility^166^, which, coupled with their ferroelectric nature, could herald game-changing advancements in integrated photonic circuits, significantly slashing fabrication costs. However, there remains ample room for improvement, particularly in optimizing film quality and enhancing ferroelectricity at thicker film dimensions, which is paramount for minimizing optical losses given their inverse relationship^167,168^.

**Fig. 18:**
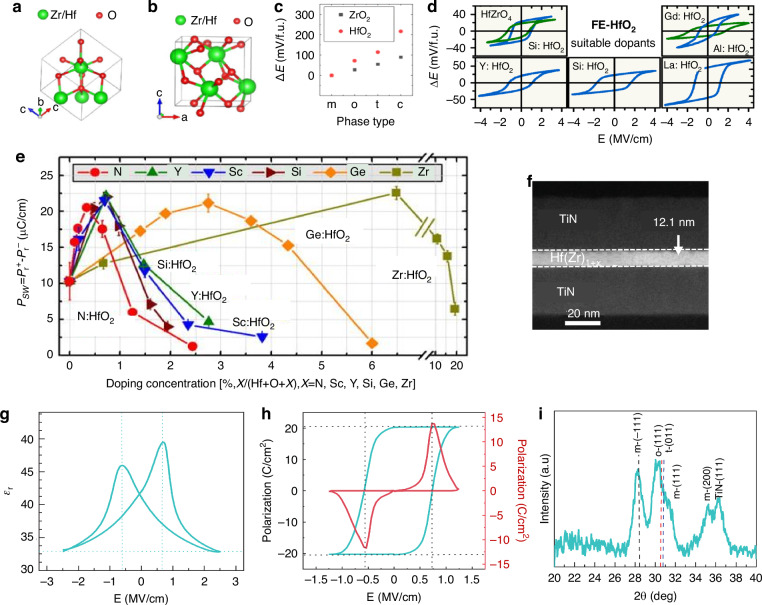
Structural and ferroelectric properties of HZO system. **a** The crystallographic structures of rhombohedral ZrO_2_-HfO_2_ (space group R3m), **b** orthorhombic ZrO_2_-HfO_2_ (space group *Pb*21). Reproduced from ref. ^177^ with permission from American Physical Society. **c** The calculated difference in total energy (Δ*E*) for the m-phase, o-phase, t-phase, and c-phase of fluorite *M*O_2_ (where *M* represents Hf or Zr) is presented. This Δ*E* is defined as the energy difference between the other phases and the m-phase, expressed in units of meV per formula unit (meV/f.u.). The green and red spheres in the representation signify Hf/Zr and O atoms, respectively. Reproduced with permission from ref. ^16^. **d** The PE-hysteresis measurements conducted on TiN/8–10 nm *X*:HfO_2_ or HfZrO_4_/TiN capacitors have unveiled ferroelectric properties for various dopants *X* incorporated at concentrations below 10 mol% in HfO_2_. Notably, a maximum remanent polarization (*P*_*r*_) of ~45 µC/cm^2^ has been achieved in thin films of La-doped HfO_2_. Reproduced from ref. ^181^ with permission from IEEE Publishing. **e** The switchable polarization (PSW) of 27 (62) nm HfO_2_ films is presented as a function of doping concentrations for various dopants, including Sc, Y, Si, Ge, Zr, and N. The doping concentration is defined as the total atomic percentage, where doping concentration = X/(Hf + O + *X*), with *X* representing the dopant atom. Reproduced from ref. ^183^ with permission from American Institute of Physics Publishing. **f** A cross-sectional STEM-HAADF image depicts the TiN/Hf(Zr)_1+x_O_2_/TiN structure, featuring a thickness of 12.1 nm, with a scale bar indicating 20 nm for reference. **g** Small-signal CV hysteresis measurements at 10 kHz (50-mV amplitude) are presented for 12-nm Hf(Zr)_1+x_O_2_ capacitors. **h** PUND analysis of Hf(Zr)_1+*x*_O_2_ capacitors under an electric field of 1 kHz frequency reveals distinct curves: the red curve represents the I-E relationship post-extraction of non-ferroelectric switching contributions, while the blue curve integrates to yield the corresponding P-E hysteresis loop. **i** GIXRD spectra are provided for TiN/Hf(Zr)_1+x_O_2_/TiN stacked capacitors, offering insights into their structural characteristics. Reproduced from ref. ^185^ with permission from American Association for the Advancement of Science

Notably, the orthorhombic *Pbc*2_1_ (o-phase), commonly observed in polycrystalline thin films, has exhibited ferroelectricity across a broad thickness spectrum, spanning from 1 nm to 1 µm^169–172^, and has also been reported in epitaxial films thinner than 20 nm^173,174^. The rhombohedral *R*3*m* (r-phase), on the other hand, has been studied within a thickness range of 5 to 40 nm^165,175,176^. Figure 18a, b illustrate the crystal structures of *R*3*m* and *Pbc*2_1_ within the rhombohedral ZrO_2_-HfO_2_ space group^177^, while Fig. 18c illustrates the free energy difference (Δ*E*) between the m-phase and other phases, calculated as Δ*E* = *E*_other-phase_–*E*_m-phase_, where the units are meV per formula unit (meV/f.u.). At room temperature, the m-phase exhibits the lowest energy, making it the most stable phase for *M*O_2_ (where *M* is Hf or Zr). Conversely, the t-phase and c-phase are only stable at extremely high temperatures. The relatively smaller Δ*E* value for the o-phase compared to the t-phase indicates a higher degree of metastability for the o-phase. Notably, among the four phases, only the o-phase possesses polar characteristics^16^.

The electrical properties of doped HfO_2_ thin films became the subject of extensive investigation. In the midst of this progression, an intermediate FE-phase was unveiled at the monoclinic-tetragonal phase boundary in Si:HfO_2_. Since then, ferroelectricity in HfO_2_ has been validated for an array of dopants, including <10 mol% Y^164^, Al^178^, Gd^179^, Sr^180^, and, La in HfO_2_ (Fig. 18d). Furthermore, owing to the disparities in free and surface energies among the polymorphs of HfO_2_ and ZrO_2_, a monoclinic-tetragonal transition, and subsequently, FE-properties, can be observed in thin films composed of HfO_2_-ZrO_2_ solid solutions as well^181,182^. In 2017, Xu et al. further reported that the P_SW_-dop% relationship of HfO_2_ can be accurately modeled as a parabolic function: ${P}_{{SW}}=-{a}_{0}{\left(\propto \times {dop} \% -{b}_{0}\right)}^{2}+{P}_{{SW}}^{{Max}}$^183^. As depicted in Fig. 18e, the *P*_*SW*_ doping percentage plot reveals that 0.34% N incorporation in HfO_2_ achieves maximum ferroelectricity, whereas, for Si, Y, or Sc incorporation, a 0.7% doping concentration is necessary. For Ge or Zr doping, even higher concentrations are required. Notably, another significant observation is that the maximum *P*_*SW*_ attained for all dopants in the study is 22 (±2) μC/cm^2^. Varying dopant sensitivities are evident in Fig. 18e. Despite the distinct roles of dopant size and *V*_*o*_ formation in HfO_2_’s ferroelectric phase, the *P*_*SW*_ -doping concentration relationship for all dopants shows a similar trend, indicating a shared mechanism for phase formation that operates regardless of *V*_*o*_ or dopant size effects^183^. In 2023, Yuan Wang’s team published a report on Planar metal-ferroelectric-metal (MFM) capacitors and the structural characterization of Hf(Zr)_1+*x*_O_2_ films, as illustrated in Fig. 18f–h. They present a cross-sectional STEM-HAADF image illustrating the stacking of TiN/Hf(Zr)_1+*x*_O_2_/TiN layers (Fig. 18f). Characteristic small-signal capacitance-voltage (C–V) curves, commonly observed in MFM capacitors, are displayed in Fig. 18g. Furthermore, Yuan Wang’s team exhibit a prototypical hysteresis polarization-electric field (P-E) loop for the TiN/Hf(Zr)_1+*x*_O_2_/TiN device after undergoing rapid thermal annealing at 55 °C in Fig. 18h, indicative of an exceptionally low coercive field (*E*_*c*_) of −0.58 MV/cm and +0.73 MV/cm. Notably, they achieved a substantial *P*_*r*_ value of 22 mC/cm^2^ at a modest driving electric field of 1.25 MV/cm. The ${\varepsilon }_{r}$ and *E*_*c*_ characteristics diverge from those exhibited by o-phase ferroelectrics, prompting us to conduct grazing incidence x-ray diffraction (GIXRD) on the Hf(Zr)_1+*x*_O_2_ film (Fig. 18i). The GIXRD results suggest that the polycrystalline sample comprises both m-phase and r-phase components. Typically, the reflection peaks of o(111) and t(011) in HZO films occur at ~30.5° and 30.8°, respectively^165,184^. However, the 2θ value for this Hf(Zr)_1+*x*_O_2_ film is slightly shifted to around 30.1°. To conclusively verify the lattice structure of the Hf(Zr)_1+*x*_O_2_ thin film, TEM analysis was deemed essential^185^.

**Fig. 19:**
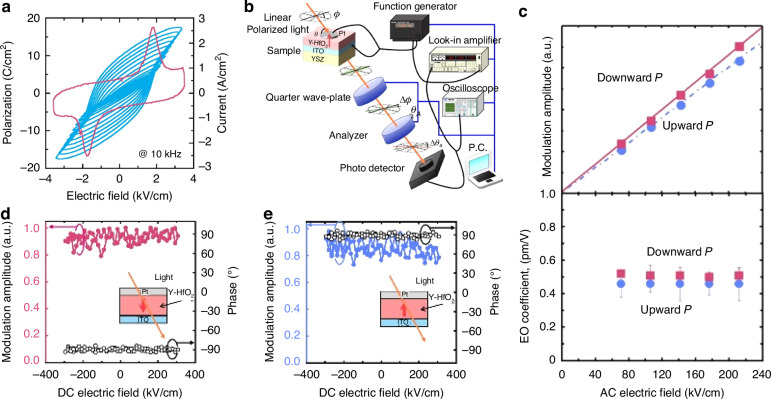
Ferroelectric and EO properties of HfO2-based thin film. **a** The polarization (depicted in blue) and current hysteresis loop (highlighted in red) were measured at a frequency of 10 kHz for the Y-HfO_2_ film. **b** A schematic representation of modulation ellipsometry applied to the Y-HfO_2_ film is presented. **c** The AC electric field’s influence on the modulation amplitude (upper graph) and the EO coefficient *r*_*c*_ (lower graph) is shown, distinguishing between cases where the spontaneous polarization points downwards (red) and upwards (blue). **d**, **e** The DC electric field-dependent modulation amplitude and phase are illustrated, considering scenarios where the spontaneous polarization is oriented downwards (**d**) and upwards (**e**). Reproduced with permission from ref. ^186^

Substantial advancements have been achieved in investigating the EO coefficient of HfO_2_. Figure 19a vividly presents the polarization-electric field (P-E) hysteresis loop of a Y-doped HfO_2_ film, meticulously measured by Shinya Kondo’s team at a frequency of 10 kHz^186^. Distinct hysteresis loops and polarization reversal currents, indicative of the ferroelectric nature of the thin film, are clearly discernible. Notably, the residual polarization of ~10 μC/cm² aligns closely with that of a Y-HfO_2_ film crafted by Reijiro Shimura’s team via RF magnetron sputtering under identical deposition conditions^172^. Conversely, an undoped HfO_2_ film fails to exhibit polarization reversal currents, pointing to the absence of ferroelectric properties, as this data is not shown here. In a bid to ascertain whether ferroelectric Y-HfO_2_ thin films exhibit linear EO effects, Shinya Kondo’s team, in 2021, adopted a modulated elliptic polarization technique, depicted in Fig. 19b. With the angle of incidence and polarization of a 632.8 nm He-Ne laser fixed at 45°, a 10 kHz sinusoidal magnetic field, augmented with DC voltage, was applied to the sample. For DC field-dependent measurements, an EAC equivalent to a peak-to-peak voltage of 35 kV/cm was utilized, while a zero-DC field was employed for AC field-dependent assessments. EO measurements ensued subsequent to the application of both positive and negative polarization biases. Figure 19c, d respectively illustrate the DC field’s influence on the Y-HfO_2_ film, with spontaneous polarization directed downwards and upwards. An almost constant amplitude, unaffected by the applied DC field, underscores a stable EO response within DC voltages beneath the coercive force field (1.81 MV/cm, refer Fig. 19a). Notably, the phases differ by 180°. These findings imply that the observed photomodulation stems from the EO response intrinsic to the ferroelectricity of Y-HfO_2_ films. Regarding AC voltage, Fig. 19e showcases the amplitude of the Y-HfO_2_ film. The modulation amplitudes, nearly identical for both positive and negative polarizations, exhibit a linear increase with the applied AC magnetic field. Consequently, the estimated effective linear EO coefficient (*r*_*c*_) remains relatively constant within the applied AC field, averaging 0.51 pm/V and 0.46 pm/V for spontaneous polarization directed downwards and upwards, respectively. Although these values lag behind those of conventional perovskite-based ferroelectrics like LiNbO_3_ (*r*_*c*_ ≈ 20 pm/V) and BaTiO_3_ (*r*_*33*_ = 105 pm/V), they surpass ZnO films with wurtzite structures by either twofold or more (0.2 pm/V)^186–189^.

### ZnO-based ferroelectrics and EO effect

**Fig. 20:**
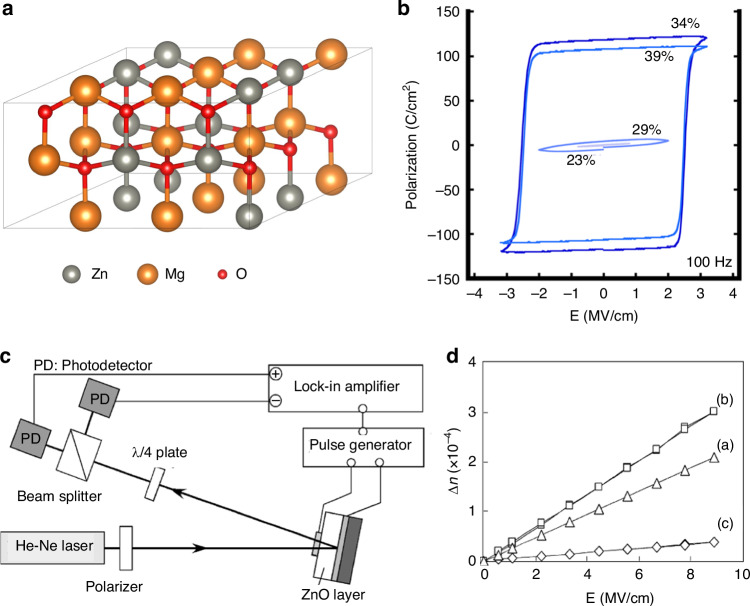
Ferroelectric and EO properties of ZnO-based thin film. **a** Schematic illustration of the 44.4% Mg-doped ZnO crystal structure. **b** Polarization hysteresis loops of ferroelectric Zn_1−*x*_Mg_*x*_O films with *x* = 0.23, 0.29, 0.34, and 0.37 Mg content. Reproduced from ref. ^161^ with permission from American Institute of Physics Publishing: Journal of Applied Physics. Schematic diagram of EO measurement system (**c**) and Δ*n* (**d**) of Mn-doped ZnO film as a function of the electric field at 100 Hz, 1 kHz, 0 kHz. Reproduced from ref. ^193^ with permission from Elsevier: Journal of Alloys and Compounds

ZnO, with its distinctive hexagonal wurtzite crystal structure, boasts unit cell parameters of *a* = 0.3253 nm and *c* = 0.5207 nm. As depicted in Fig. 20a^190^, the crystal structure is characterized by alternating layers of oxygen and zinc atoms stacked in an ABAB sequence, each layer forming a (001) crystal plane. This unique arrangement imparts ZnO with both an oxygen- and a zinc-polarized surface, influencing its optical, electrical, and thermal stability properties, as well as its tendency to incorporate impurities. Notably, the (001) surface of ZnO exhibits smoothness in equilibrium, and during film growth, a strong preference for the (001) or c-axis orientation is observed, commonly referred to as the c-axis preferred orientation. The Mg-doped ZnO (Zn_1−*x*_Mg_*x*_O) film exhibits ferroelectric properties to a specific extent, with its hysteresis loop illustrated in Fig. 20b^161^.

ZnO stands as a pivotal multifunctional crystalline material, renowned for its low deposition temperature, high melting point, minimal electron-induced defects, and straightforward preparation process. Its versatility has led to widespread adoption across diverse fields for over half a century. Silicon-integrated ZnO films offer seamless compatibility with conventional silicon-planar technologies, pioneering avenues for optoelectronic device fabrication. Furthermore, ZnO thin films, renowned for their high resistivity and uniform c-axis orientation, possess exceptional piezoelectric constants and electromechanical coupling coefficients, rendering them suitable for piezoelectric, calendaring, electroacoustic, and acousto-optic devices^191^.

Despite the long-standing reports on the EO effect of ZnO bulk crystals, its performance falls short compared to ferroelectric materials like PZT. Hu et al. displayed a calibrated linear EO coefficient of merely 1.30 pm/V for ZnO^192^. However, by introducing Li, Mg, or other dopants, ZnO can exhibit ferroelectric properties. As shown in Fig. 20c, Nagata et al. delved into the EO behavior of ZnO:Mn films, discovering that manganese doping not only mitigated leakage current issues but also imparted a linear EO response with a coefficient of 0.5 pm/V in dipole-polarized ZnO:Mn films, surpassing that of pure ZnO Fig. 20d^193^. Additionally, Dabir et al. studied Al and Cu-doped ZnO films prepared via the sol-gel method and found that both dopants, while not significantly different from each other, enhanced the EO properties of ZnO films^194^. Furthermore, ZnO’s exceptional ultraviolet emission properties, stemming from its wide direct bandgap and substantial exciton binding energy, facilitate its potential to integrate laser generation and modulation within a single microcavity as an EO material. To substantiate this capability, Li et al. conducted an experiment in 2022, utilizing high-quality, smoothly surfaced ZnO hexagonal micro rods as ultraviolet laser microcavities^195^. Experiments varying electric fields relative to a crystal’s c-axis showed linear and quadratic refractive index changes, indicating achievable EO modulation in laser cavities. This discovery facilitates the integration of UV laser generation and dynamic modulation, advancing optoelectronic and photonic technologies. In 2023, Hajer Saadi and her team optimized the EO properties of ZnO crystals by fine-tuning the Co doping level, which significantly enhanced the dielectric coefficient and, in turn, improved the energy storage performance of the co-doped ZnO nanopowders^196^. Upon investigating the EO effect of ZnO, it becomes evident that despite its significant ferro-polarization, the EO effect remains minimal. This is attributed to the lack of clarity surrounding the mechanism of the ferroelectric origin in ZnO. Consequently, further research is imperative to unravel strategies for regulating and enhancing the EO coefficient of ZnO.

### AlN-based ferroelectrics and EO effect

**Fig. 21:**
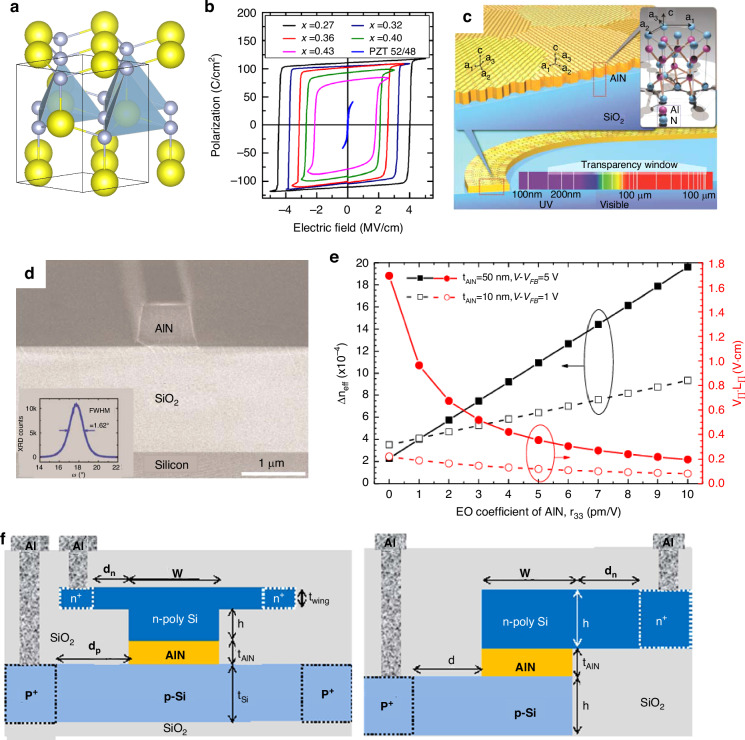
Ferroelectric and EO properties of AlN-based thin film. **a** Crystal structure of AlN. **b** The *P-E* ring of ferroelectric Al_1-*x*_Sc_*x*_N has a Sc content of *x* = 0.27, 0.32, 0.36, 0.40, and 0.43 and PZT 52/48. Reproduced from ref. ^163^ with permission from American Institute of Physics Publishing: Journal of Applied Physics. **c** The sputtered AlN thin films, highlighted in yellow, display a polycrystalline nature, comprising columnar micrograins with a c-axis orientation perpendicular to the film plane. The in-plane orientations of these micrograins are randomized, as depicted by the hatched lines on the surface. Inset: The atomic arrangement within a hexagonal AlN unit cell is illustrated. AlN’s exceptional ultrawide bandgap of 6.2 eV enables an optical transparency window that spans from UV to infrared regions. **d** A cross-sectional SEM image reveals a patterned AlN waveguide resting on a 2 μm thick layer of thermally grown SiO_2_ atop silicon substrates. The inset showcases a representative X-ray rocking curve measurement of the AlN (0002) peak, with a full width at half-maximum (FWHM) of ~1.62°, signifying a high-quality c-axis orientation. Reproduced from ref. ^199^ with permission from American Chemical Society Publications. **e** Δn_eff_ and V_π_⋅L_π_ as a function of the EO coefficient of the modulator gate dielectric. **f** Schematic cross-sectional diagram of two typical Si MOS capacitor phase modulators. Reproduced from ref. ^200^ with permission from IEEE Publishing

AlN and ZnO are esteemed direct bandgap semiconductor materials characterized by substantial bandgap widths, along with being lead-free, environmentally benign, and cost-effective. Notably, AlN surpasses ZnO in its compatibility with CMOS processes and boasts a superior sound propagation velocity. The prevalent stable configuration of AlN is the hexagonal wurtzite structure, exemplified in Fig. 21a, where lattice constants a and c are 0.311 nm and 0.498 nm, respectively^197^. This unit cell hosts two distinct chemical bonds: the polar covalent B1 bond and the coordination B2 bond, forming angles of 107.7° and 110.5°, respectively. AlN’s asymmetrical crystal structure endows it with piezoelectricity, nonlinear properties (Pockels effect and second-order nonlinearity), and pyroelectricity. Nonetheless, Scandium (Sc) doping can significantly enhance the electromechanical coupling coefficient and piezoelectric response speed of AlN thin films, underscoring the importance of investigating the growth, quality of AlScN thin films, and the potential for reconfigurable photonic integrated circuits as key research objectives in related fields moving forward^198^. Furthermore, Sc-doped Al_1-*x*_Sc_*x*_N demonstrates commendable ferroelectric properties, as evidenced by the P-E (polarization versus electric field) hysteresis loops presented in Fig. 21b for various Sc concentrations of *x* = 0.27, 0.32, 0.36, 0.40, and 0.43^163^.

Upon optimization, sputter-deposited aluminum nitride (AlN) films exhibit a distinct columnar micrograin structure, featuring a c-axis orientation (0002) perpendicular to the film plane, as illustrated in Fig. 21c. This preferred orientation is pivotal for maximizing the exploitation of AlN’s *χ*^(2)^ tensor component (*d*_*33*_). The quality of this c-axis orientation is quantitatively assessed through X-ray diffraction rocking curve measurements of the AlN (0002) peak. As evident from the inset in Fig. 21d, our AlN films exhibit a typical full width at half-maximum of <2°, signifying a highly oriented film. Additionally, AlN boasts residual stress as low as ±75 MPa, which is significantly lower than that of silicon nitride, enabling the production of micrometer-thick films and facilitating the fabrication of highly confined waveguides in both TE and transverse magnetic polarizations^199^.

Zhu et al. innovatively designed a silicon MIS phase modulator utilizing AlN thin films as gate dielectrics^200^. As shown in Fig. 21e, f, this design harnesses the AlN gate dielectric’s inherent bubblers effect, augmenting phase modulation beyond the free carrier plasma dispersion effect of Si. Notably, they measured an EO coefficient of ~1 pm/V for AlN, albeit lower than that of EO materials like lithium niobate, yet this facilitates improved modulation efficiency without compromising other performance metrics. Furthermore, AlN exhibits pronounced second-order nonlinearity and boasts deposition techniques compatible with a diverse array of substrates, silicon included. Xiong et al. capitalized on these strengths, crafting AlN waveguides on insulators with minimal propagation losses (0.6 dB/cm), leveraging CMOS-compatible sputtered films. They leveraged AlN’s intrinsic Pockels effect to demonstrate EO modulation speeds of up to 4.5 Gb/s, while achieving an impressive power consumption of merely 10 fJ/bit^199^. Similarly, Zhu et al. achieved a remarkable ultra-low-loss AlN single-mode channel waveguide, exhibiting propagation losses of ~0.42 dB/cm at 1550 nm and 2.36 dB/cm at 905 nm^201^.

Moreover, AlN stands out as one of the semiconductors with the widest bandgap (6.2 eV), enabling it to operate across a broad spectrum, spanning from ultraviolet to infrared wavelengths. Notably, its low propagation loss in the mid-infrared region holds promise in addressing the scarcity of advanced EO modulators within this wavelength range. Leveraging this unique feature, Liu et al. groundbreakingly demonstrated in 2016 the first CMOS-compatible mid-infrared EO modulator. By applying a voltage of 15 V to the modulator featuring a slotted waveguide structure, they achieved a substantial effective waveguide index change exceeding 2 × 10^−^^5^, while maintaining a reasonable transmission loss of ~2 dB/cm at a wavelength of 2.5 μm^202^. Their findings underscore the efficacy of AlN materials in harnessing the EO effect across the expansive mid-infrared wavelength spectrum. Moreover, the lower dielectric constant of AlN compared to ZnO theoretically enables the achievement of an exceptionally high bandwidth, potentially surpassing even lithium niobate. Despite AlN’s significant ferroelectric polarization, its measured EO coefficient remains low, necessitating further research into its underlying mechanisms to effectively optimize and control its EO response, akin to ZnO. This endeavor is crucial for laying the groundwork for potential future applications.

## Mechanism of EO effect in ferroelectrics

Research on the mechanism of EO effects aims to clarify the essential correlation between electronic and light coupling in EO materials. It is an inevitable path to search for and explore high-performance next-generation EO materials. By establishing a quantitative relationship model among refractive index, ferroelectric polarization, and external electric field, it is possible to effectively design and prepare high EO coefficient thin films, such as lattice orientation, ferroelectric polarization strength, and direction, the direction of the external electric and optical field, device fabrication.

Notably, a sufficiently strong external electric field can induce phase changes in substances through melting or crystallization, but this does not constitute an EO effect. EO effects exclusively influence material polarization, not altering the crystal structure permanently. The material’s polarization magnitude can be formulated as a function of the external field’s strength^203^. The Pockels effect, discovered by Pockels in 1893, manifests in asymmetrical central crystals like BTO, where the refractive index varies linearly with the applied electric field. In contrast, the Kerr effect, discovered by Kerr in 1875, occurs in all transparent media, exhibiting a linear relationship between the refractive index and the square of the applied electric field. Although the definition of the EO coefficient and its relationship with ferroelectric polarization are relatively clear, there are still difficulties in establishing deeper physical mechanism models and quantitative relationships. The important ones come from several aspects:

Firstly, in terms of structural representation, the current EO mechanism involves ferroelectric polarization and optical coupling. General structural characterization can only represent the state after the application of an electric field and the change in domain structure, but it cannot detect the polarization response dynamics of the optical frequency polarization unit within the lattice under the domain structure state, because the optical frequency has already exceeded the limit of the current time-space resolution projection electron microscopy equipment. Therefore, the direct characterization of the ultrafast dynamics of the ferroelectric polarization microstructure at optical frequencies is a very challenging problem for the future.

**Fig. 22:**
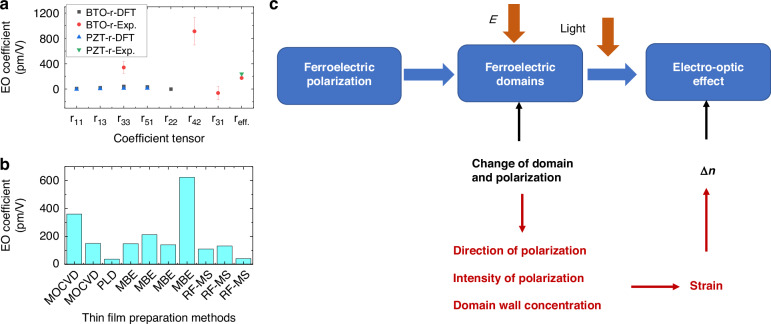
Variations of EO coefficients in ferroelectric materials. **a** Comparison of the EO coefficient values of BTO and PZT. The values were obtained from theoretical calculations and experimental testing^8,9,67^. **b** Differences in EO coefficients of BTO under different preparation methods^8,127–135,142,143^. **c** Illustration of the correlation between ferroelectric polarization and electro-optic effect

Secondly, in terms of the dynamic mechanism, the primary reliance is still on first-principles calculations and phase-field simulations. Current first-principles calculations can compute the saturation polarization, phonon vibration frequency spectrum and modes, and optical frequency refractive index. Although it is possible to calculate the EO coefficient separately by computing electronic polarization and lattice phonon vibration, the calculated values often differ significantly from experimental values. Figure 22a, b shows the selected values reported by literature. Theoretical calculations for BTO indicate that its EO coefficient *r*_*33*_ is the largest, reaching ~45 pm/V^65^, while experimental measurements have yielded values as high as 340 pm/V^8^, almost an order of magnitude difference. Some experimental reports suggest a comprehensive effective EO coefficient as high as 175 pm/V. The same phenomenon occurs with PZT, where the calculated *r*_*51*_ is 13^65^, but the effective EO coefficient obtained from experimental testing is as high as 240 pm/V^9^. One main reason is that the first-principles calculations do not take into account the converse piezoelectric effect, which involves electron-phonon coupling. Additionally, calculating the refractive index changes under the application of an electric field is also a challenge, which is generally estimated by calculating the energy based on the reversal changes of different polarization states and then converting it into an electric field. As shown in Fig. 22c, the EO effect underscores a profound interplay with domain structure and polarization control, revealing it as a complex, cross-scale dynamic process governing the regulation and response of polarization structures. This process encompasses the intricate coupling and dynamic behavior across multi-level, cross-scale structures, thereby accounting for the inherent challenges in achieving precise theoretical calculations. Furthermore, the intertwined nature of electricity and light within the applied electric field, coupled with the electric domain structure, polarization direction, and light field direction, significantly contributes to the significant variation in EO coefficients observed across diverse preparation methods. Therefore, in the future, it is necessary to correct the method of calculating the EO coefficient by first-principles calculations to improve accuracy. Phase-field simulation calculations depend on first-principles calculations or experimentally provided boundary conditions^204^. Although they can compute the domain structure under the influence of an electric field^205^, the calculated EO coefficients also differ from the actual values. The main reason is that the calculations do not consider electronic polarization and electron-phonon coupling. At the same time, the current calculations cannot obtain more accurate free energy, which leads to deviations in the calculated EO coefficients^206^. In the future, phase field simulations also need to improve their accuracy.

Finally, in addition to the difficulty in characterizing and accurately theoretically analyzing the dynamic behavior of microstructural coupling, the system itself is complex, encompassing lattice, defect dipoles, ferroelectric polarization phases, ferroelectric domain structures, heterointerfaces, and other multi-level structural factors that have an influential and decisive role in polarization^207^. Under the action of an electric field, these multi-level structures couple with each other, complicating the research on the correlation mechanism between ferroelectric polarization and microstructure, thereby increasing the difficulty of studying the micro-mechanism. This cross-scale high-frequency dynamic behavior poses a challenge to existing theoretical analysis and analytical methods.

## Conclusion and prospect

### Summary

In conclusion, the evolution of mobile communication, particularly 5 G, is inextricably linked to the advancements in optical communication technology. EO modulation has emerged as the cornerstone modulation technique for light waves in fiber-optic communication, owing to its unparalleled modulation speed and minimal loss. As future scientific and technological advancements accelerate, the quest for swifter and more capacious data transmission across various sectors intensifies, prompting heightened expectations for the speed and capacity of optical communication systems. This, in turn, necessitates the development of EO materials endowed with substantial EO coefficients, exceptional light transmittance, and stable physicochemical properties, as well as EO modulators capable of faster modulation, reduced loss, and smaller form factors. In this paper, we delve into inorganic optic materials, comprehensively reviewing the research endeavors on space EO modulation materials and integrated EO films, grounded in the fundamental principles of EO effect and modulation. Furthermore, we undertook an in-depth analysis of the basic ferroelectric properties and EO effects exhibited by recently discovered, CMOS-compatible, non-perovskite inorganic ferroelectric materials. The specific research contents and conclusions are as follows:

Currently, LNO-based modulators dominate the high-speed EO modulator market. However, their limited EO coefficient constrains the performance of commercial modulators utilizing bulk lithium niobate, resulting in larger sizes, higher driving voltages. The development of inorganic optical materials for next-generation EO materials especially for silicon-chip-level integration would be expected to focus on BTO and PZT that exhibit much higher EO coefficients.

Various factors, including temperature, domain structure, and manufacturing process, exert a profound influence on the EO effect. For instance, as the temperature rises, the secondary EO coefficient of PMN-PT ceramics diminishes, whereas the linear EO coefficient of PZT films undergoes a gradual increase. The dimensions of the ferroelectric domain in PMN-PT ceramics directly impact their EO effect; domains that are too small prolong the growth process, whereas overly large domains exhibit reduced responsiveness to applied electric fields, both scenarios weakening the EO effect. Similarly, the domain structure of BTO films also modulates their EO properties; augmenting the DC bias in multi-domain BTO films can amplify their EO response, with the EO coefficients varying significantly based on the relative orientation between the electric field and domain direction. Regarding preparation methods, molecular beam epitaxy yields BTO films with the most pronounced EO coefficient, while magnetron sputtering offers a faster growth rate, making it a more viable option for industrial-scale production.

In the foreseeable future, ferroelectric materials in the realm of inorganic electro-optics promise vast developmental potential, with novel ferroelectric EO modulators poised to dominate both research endeavors and market trends. Additionally, traditional bulk crystals have fallen short of contemporary demands, underscoring ample scope for the advancement of thin-film EO materials. Intensified research efforts are imperative to enhance the cost-effectiveness of these thin-film materials, further fueling their progress.

### Challenge

Although BTO and PZT have EO coefficients an order of magnitude higher than LN, making them promising next-generation chip-level materials, their complex microstructures and polarization characteristics present challenges in studying their EO modulation mechanisms. For example, how can we more accurately obtain their EO coefficients through theoretical calculations or in-situ simulations? How can we create a quantitative model linking the multi-level structural dynamics of ferroelectric materials to their EO coefficients or refractive index via experiments, calculations, and microstructural analysis? This model will crucially inform the design of EO coupling in modulators and device fabrication.

In addition to the emergent EO materials with high Pockels coefficients, in the era of 5 G, EO modulators face formidable challenges to cater to the diverse requirements of fields like optical communication and optoelectronic technology. When an electric field is applied to an EO crystal, it triggers alterations in the crystal’s refractive index, subsequently modifying the phase, amplitude, intensity, and polarization state of light waves traversing it. EO modulators adeptly detect and amplify these changes via sophisticated optical components, ultimately facilitating the modulation of optical signals. Within these modulators, the input light wave undergoes transformations in its phase, amplitude, and other parameters upon encountering the electric field-influenced EO crystal. These modified light waves then undergo further optical processing (e.g., interference, coupling) to yield the desired modulation signal. Notably, in an MZ interferometer setup, the modulation is achieved by manipulating the phase difference between two interfering light beams. Consequently, high-performance EO modulators necessitate materials with superior EO properties, encompassing a high EO coefficient, broad modulation bandwidth, robust thermal stability, and minimal losses. Despite significant advancements in EO material research, prevalent materials still exhibit limitations; for instance, LNO suffers from substantial losses and intricate preparation processes, while BTO has a low secondary EO coefficient and a temperature-sensitive dielectric constant. In the future, it is imperative to intensify research efforts aimed at refining the manufacturing processes of existing EO materials and enhancing their performance capabilities. Furthermore, we eagerly anticipate the emergence of novel EO materials that will broaden our horizons in this field.

### Prospect

Based on the comprehensive research presented in this paper, there are several avenues worthy of further exploration:

1. Our review of the advancements in perovskite-based ferroelectric EO materials alongside emergent CMOS-compatible inorganic non-perovskite counterparts highlights a prevailing focus on ferroelectric and EO modulators. However, a notable performance gap persists when comparing these findings to the benchmark applications of LNO and similar materials. Consequently, future research endeavors in inorganic ferroelectric EO modulation materials should prioritize elucidating the intricate interplay between ferroelectric polarization and the EO effect, ultimately aiming to optimize the latter through a deeper understanding of the underlying mechanisms.
2. This paper delves into the research advancements of inorganic optical materials. Specifically, we propose two primary directions: Firstly, conducting simulation-based studies to meticulously design the parameters of the response EO modulator, thereby optimizing the device’s structure. Secondly, thoroughly investigating and validating the optical transmission loss, device insertion loss, and stability characteristics of these inorganic materials. Ultimately, our aim is to identify and explore the next-generation inorganic EO materials that hold the greatest potential to emerge as viable alternatives to LNO.

**Fig. 23:**
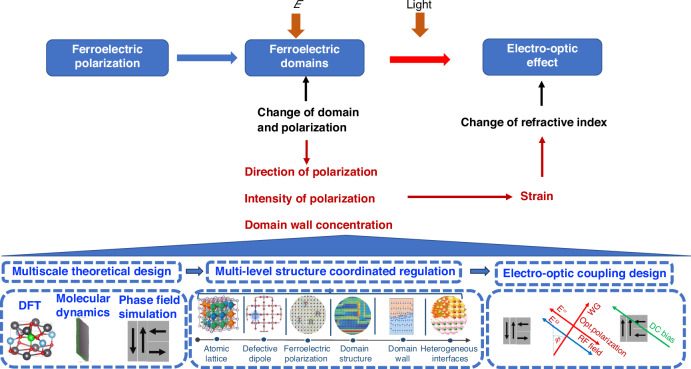
Proposed approach for future research on EO mechanism and property optimization of inorganic ferroelectrics. The multiscale structure picture and EO coupling design illustration were referenced from the literature. Reproduced from ref. ^207^ with permission from Royal Chemistry Society: Materials Horizons

As shown in Fig. 23, the findings suggest that the ferroelectric EO modulation effect is intricately tied to domain structure and polarization regulation, constituting a dynamic, cross-scale process that governs the regulation and response of polarization structures. To unravel the underlying mechanisms and inform material design, future research must integrate theoretical frameworks, computational methods such as DFT, molecular dynamics, phase field simulations, and material design strategies. Additionally, a comprehensive analysis of the material’s multi-level structure and coupling behavior offers insights into how these structures can be leveraged synergistically to modulate both iron chain arrangement and refractive index. Ultimately, by harmoniously integrating the polarization direction, electric field direction, light field direction, and co-design principles, the EO modulation effect can be optimized, thereby guiding the development of advanced devices.
